# Heterogeneity and interplay of the extracellular vesicle small RNA transcriptome and proteome

**DOI:** 10.1038/s41598-018-28485-9

**Published:** 2018-07-17

**Authors:** Helena Sork, Giulia Corso, Kaarel Krjutskov, Henrik J. Johansson, Joel Z. Nordin, Oscar P. B. Wiklander, Yi Xin Fiona Lee, Jakub Orzechowski Westholm, Janne Lehtiö, Matthew J. A. Wood, Imre Mäger, Samir EL Andaloussi

**Affiliations:** 1 Department of Laboratory Medicine, Karolinska Institutet, Huddinge, Sweden; 2 Competence Centre on Health Technologies, Tartu, Estonia; 3 Department of Biosciences and Nutrition, Karolinska Institutet, Huddinge, Sweden; 4 University of Helsinki and Folkhälsan Institute of Genetics, Helsinki, Finland; 5 Science for Life Laboratory, Department of Oncology-Pathology, Karolinska Institutet, Solna, Sweden; 6 Evox Therapeutics, King Charles House, Oxford, United Kingdom; 7 Department of Physiology, Anatomy and Genetics, University of Oxford, Le Gros Clark building, Oxford, UK; 8 Science for Life Laboratory, Department of Biochemistry and Biophysics, Stockholm University, Solna, Sweden; 9 Institute of Technology, University of Tartu, Tartu, Estonia; 10 Present Address: Genome Institute of Singapore, Singapore, Republic of Singapore

## Abstract

Extracellular vesicles (EVs) mediate cell-to-cell communication by delivering or displaying macromolecules to their recipient cells. While certain broad-spectrum EV effects reflect their protein cargo composition, others have been attributed to individual EV-loaded molecules such as specific miRNAs. In this work, we have investigated the contents of vesicular cargo using small RNA sequencing of cells and EVs from HEK293T, RD4, C2C12, Neuro2a and C17.2. The majority of RNA content in EVs (49–96%) corresponded to rRNA-, coding- and tRNA fragments, corroborating with our proteomic analysis of HEK293T and C2C12 EVs which showed an enrichment of ribosome and translation-related proteins. On the other hand, the overall proportion of vesicular small RNA was relatively low and variable (2-39%) and mostly comprised of miRNAs and sequences mapping to piRNA loci. Importantly, this is one of the few studies, which systematically links vesicular RNA and protein cargo of vesicles. Our data is particularly useful for future work in unravelling the biological mechanisms underlying vesicular RNA and protein sorting and serves as an important guide in developing EVs as carriers for RNA therapeutics.

## Introduction

Extracellular vesicles are important mediators of cell-to-cell communication. These lipid bilayer vesicles of around 30–2000 nm in diameter are secreted by virtually all types of cells either constitutively or upon specific stimuli. The biogenesis of EVs includes a range of complex mechanisms, which in itself could contribute to disparate sorting of vesicular cargo and thereby support cell type specific biological effects of the vesicles. Based on their biogenesis pathways, there are three broad types of EVs - exosomes, microvesicles, and apoptotic bodies, each of those being highly heterogeneous in their own. Exosomes are intraluminal vesicles of the multivesicular body (MVB) that are released from the cell upon MVB fusion with the cell membrane, a process which is largely regulated by ESCRT proteins, Rab GTPases, and neutral sphingomyelinase^1^. Microvesicles and apoptotic bodies are both formed by outward budding of the cell membrane^2,3^. While microvesicles reflect the composition of the cytosol, apoptotic bodies can also contain cytoplasmic organelles and/or nuclear fragments^3^. Though both vesicle types tend to be larger than exosomes, the inherent heterogeneity of all populations stresses the need that biogenesis mechanisms should rather be used as the basis for their primary distinction^4^. In this study the term ‘extracellular vesicles’ is used to refer to an exosome-enriched pool of vesicles.

EVs mediate their native biological effects by transferring or displaying their cargo to target cells, whereas the effects are either related to their overall complex cargo signature, or to certain individual biologically active macromolecules. Some EV effects have been attributed to their specific miRNA content (reviewed in^5^), which can either reflect that of their cell of origin or display enrichment of certain miRNA subsets^6^. Which miRNAs are enriched in EVs often depends on the cell type and its physiological status^7^, yet miRNA traits such as DICER independence, presence of specific nucleotide sequences or -interaction with certain RNA binding proteins seem to promote the sorting of a subset of miRNAs into EVs (reviewed in 7).

The biologically active transfer of vesicular miRNAs has been demonstrated in numerous early studies^8–12^, reviewed elsewhere^5,13^ and an exponential growth in the number of new reports can be seen^14–18^. Nevertheless, miRNAs represent only a fraction of all RNA species found in EVs, with the bulk miRNA secretion being independent of vesicular material^19,20^. In addition, other species, such as tRNA, ribosomal RNA, vault RNA and Y RNA have much higher levels in EVs than an average miRNA^21–25^. Of note, the richness of the RNA repertoire in EVs is consistent with the high number of RNA binding proteins found in EVs, often being overrepresented or enriched compared to some other protein classes^26–28^.

In order to fully exploit the potential of basic biological processes for therapeutic- and diagnostic purposes, a better understanding of vesicular cargo sorting and interplay with the proteome needs to be achieved. EV-loaded miRNAs have recently gained much attention both for understanding EV biology as well as for developing EVs as biotechnological tools or therapeutic RNA nanocarriers. To shed light onto the interplay of bioactive macromolecules, we analysed the small RNA transcriptome of cells and EVs by next generation sequencing as well as investigated RNA/miRNA binding proteins in EVs with respect to the discovered EV RNA species. A thorough understanding of the synergy between these different EV bioactive macromolecules aids to develop novel EV RNA therapeutics by taking advantage of underlying cargo sorting mechanisms.

## Results

### EV characterization

To investigate the RNA and protein composition in EVs, we chose vesicles and parental cells of mouse or human origin from five cell lines, which are commonly used for EV production and/or are of specific interest due to their involvement in neurodegenerative disorders. Hence, the present analysis includes parental cells and/or EVs from HEK293T (HEK; human embryonic kidney cells), RD4 (human skeletal muscle cells), Neuro2a (neuroblastoma cells), C17.2 (immortalised mouse neural progenitor cells) and C2C12 (immortalized mouse myoblasts) cell lines.

**Figure 1:**
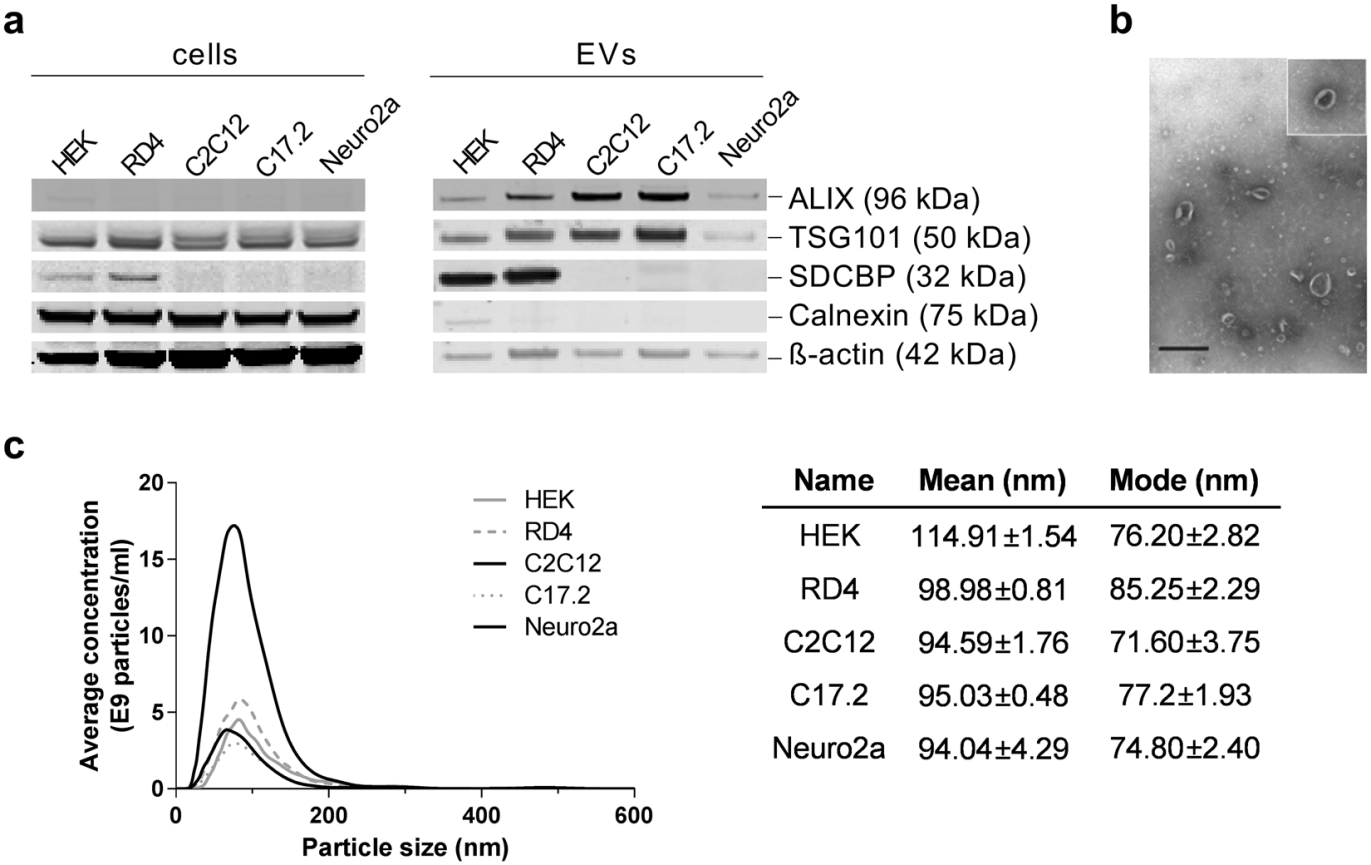
Characterization of extracellular vesicles (EVs). **(a–c)** EVs were characterized by Western blotting (5 × 10^9^ particles loaded per well) **(a)**, electron microscopy (HEK, scale bar = 500 nm) **(b)** and Nanoparticle Tracking Analysis (NTA) **(c)**, confirming the presence of ALIX, TSG101, SDCBP (syntenin, human reactive antibody) and cup-shaped morphology of particles. ß-actin serves as a loading control for cell samples. Full-length western blots can be found in Supplementary Figure S1. Mean/mode size (nm) ± SEM for NTA measurements are depicted.

EVs were characterized by Nanoparticle Tracking Analysis, transmission electron microscopy and western blot, confirming their size around 100 nm, presence of cup-shape morphology and the enrichment of ALIX and TSG101 throughout the tested samples (Fig. 1, Supplementary Figure S1).

### Small RNA sequencing analysis of parental cells and EVs

The parental cells as well as EVs were subjected to next generation sequencing followed by data analysis of the vesicular- and parental cell derived short RNAs. Due to our specific interest in vesicular small RNAs, short RNA centred size-selection of the libraries and length-restricted data analysis in the size range of 17–35 nucleotides was carried out by using a custom in-house workflow. Here we performed sequential annotation discriminating between ‘small RNA’, ‘rRNA’ and ‘other RNA’ categories (Supplementary Figure S2 and Supplementary Table S1). This length-restricted analysis was applied to specifically detect miRNAs, piRNAs as well as snRNA- and snoRNA fragments, which have previously been reported in EVs^22,25,29^.

#### EVs are relatively depleted in ‘small RNAs’

**Figure 2:**
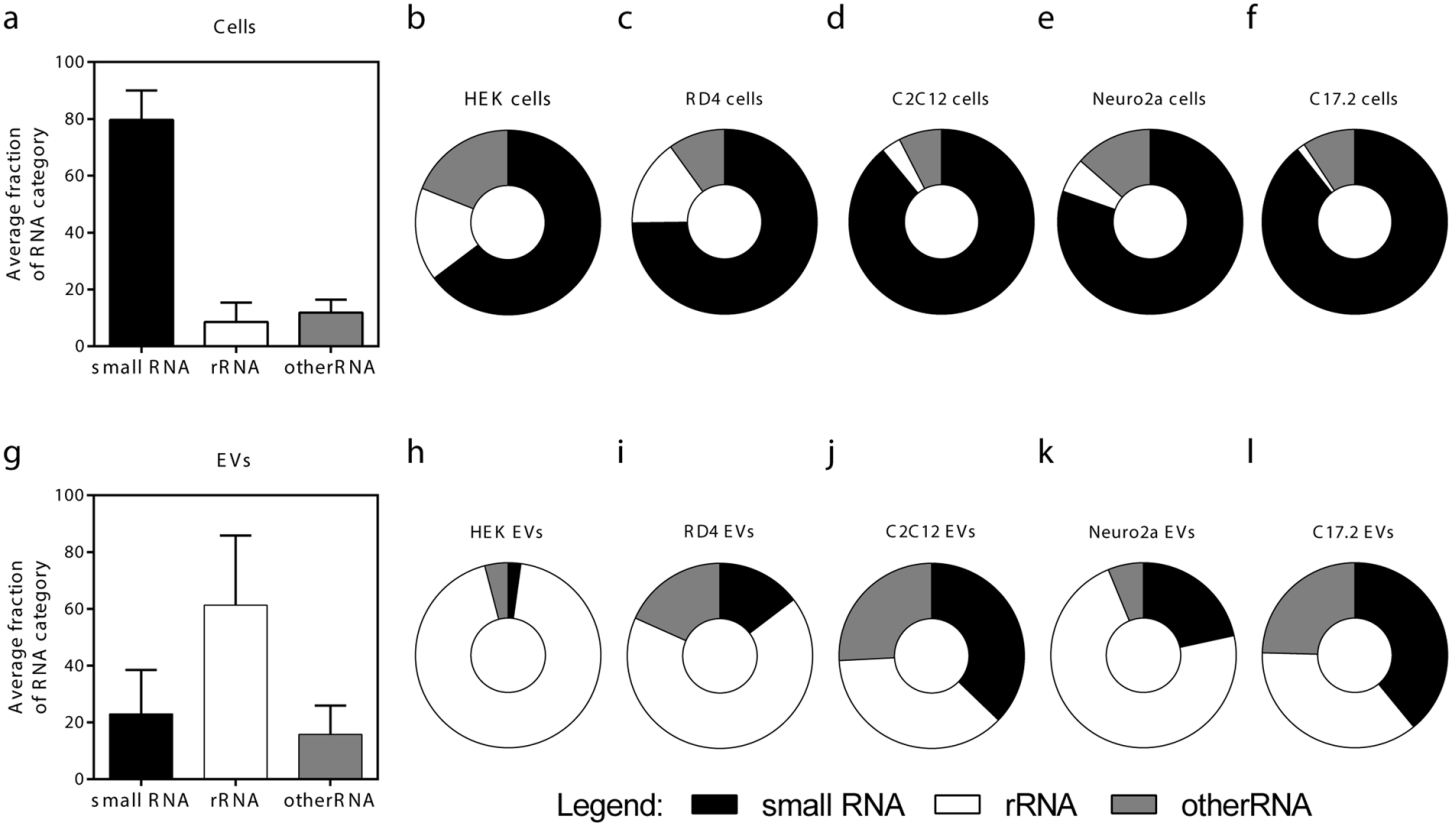
Contribution of ‘small RNAs’, ‘rRNAs’ and ‘other RNAs’ to the total pool of annotated RNA sequences. (**a–f**) EV source cells were rather enriched in small RNAs (65–89% of annotated reads) holding in average only 12% rRNA sequences. **(g–l)** In contrary, despite size-selection and data filtering excluding reads outside the 17–35 nt size range, EV samples were relatively depleted of small RNAs and instead contained ample amount of fragments derived from rRNA sequences covering up to 94% of all annotated RNAs (HEK EVs). Mean ± SD depicted. The presented categories include microRNA, piwi-interacting RNA, small nuclear RNA and small nucleolar RNA (’small RNA’ category); large and small subunit ribosomal RNA and mitochondrial ribosomal RNA (’rRNA category’); transfer RNA, mitochondrial tRNA, protein coding genes, long noncloding RNA, miscellaneous RNA, processed transcripts, pseudogenes and small cytoplasmic RNA (’other RNA’ category). Details about the subdivision can also be found in Supplementary Figure S9 and Supplementary Table S1.

Firstly, we analysed the fraction of ‘small RNA’, ‘rRNA’ and ‘other RNA’ categories within EV- and cell samples. Expectedly, in EV source cells, approximately 80% of the reads were annotated as ‘small RNA’ in accordance with the small RNA-centred size selection of the libraries and length-restricted data analysis. The level of long RNA fragments was low (average fraction of 8% and 12% for ‘rRNA’ and ‘other RNA’ reads, respectively) (Fig. 2A). Inversely, in EVs, the proportion of reads annotated as ‘small RNA’ was on average only 22% while rRNA fragments constituted on average 60% of all annotated reads (Fig. 2G), clearly highlighting an increase in the proportion of RNA fragments as compared to their parental cells.

Additionally, the annotated RNA species across EV samples were also more variable than across source cells (Fig. 2B–F,H–L). For example, in HEK EVs 94% of reads mapped to ‘rRNA’ loci and only 2% to ‘small RNA’, whereas nearly 40% of the reads in C2C12 and C17.2 EVs belonged to one of the ‘small RNA’ gene biotypes (Fig. 2B,D,F). Again, it should be emphasized that the detected ‘rRNA’, ‘other RNA’ and certain subtypes of ‘small RNA’ annotations (snRNA and snoRNA) correspond to fragments derived from these RNA species, since the data has undergone filtering, excluding reads outside of the 17–35 nucleotide size range.

#### miRNAs represent a relatively small proportion of ‘small RNAs’ in EV samples

**Figure 3:**
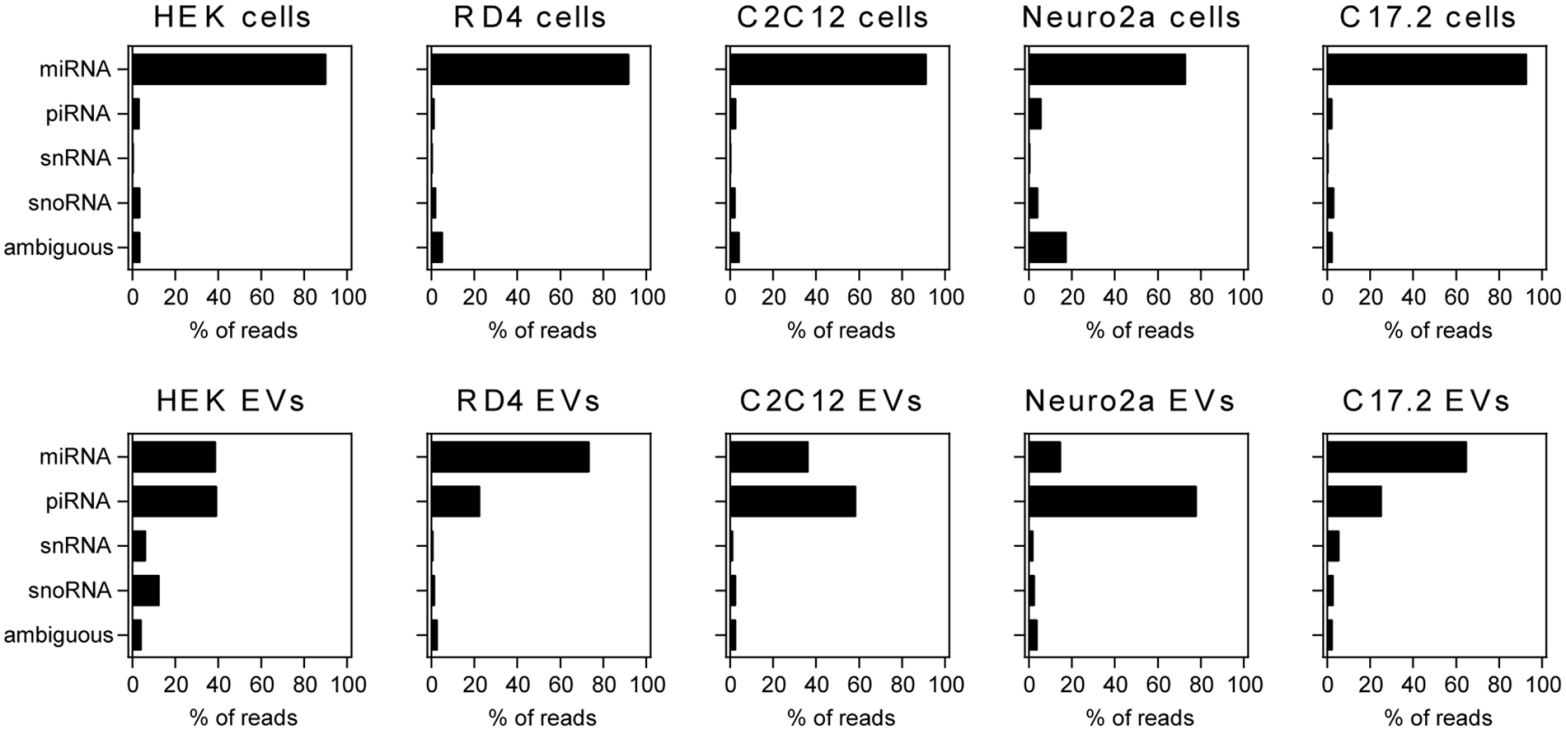
Contribution of individual gene biotypes to the ‘small RNA’ category. Within the ‘small RNAs’, the cells were (without exception) found to be enriched in miRNAs. Compared to cells, EV samples had a lower and more variable miRNA content and a higher amlount of reads annotating to piRNA loci. % of reads is denoted as the fraction of individual biotypes within the indicated category. Additional data on ambiguous annotation of piRNA/tRNA sequences is addressed in Supplementary Figure S5. Additional data on ‘rRNA’ and ‘other RNA’ categories can be found in Supplementary Figure S6.

Focusing on the gene biotypes in the ‘small RNA’ category, the majority of reads (58–83%) in all tested parental cell types could be attributed to miRNA genes with very low levels of reads annotating to piRNA loci (Fig. 3). However, in EV samples as compared to cells there was an increase in the proportion of reads mapping to piRNA loci (mean 33% *versus* 3%) and a reduction in miRNA annotations (mean 25% *versus* 77%) (Fig. 3, lower panels). Practically all vesicular miRNA reads (96–98%) fell under a size range of 20–25 nucleotides and the overall number of miRNAs in EVs was found to be consistent across all samples (Supplementary Figure S3). Though HEK EVs had the lowest proportion of small RNAs, 263 distinct miRNA annotations (read count ≥3) were detected, which was comparable to other EV samples having between 274–396 unique miRNA identifications.

**Figure 4:**
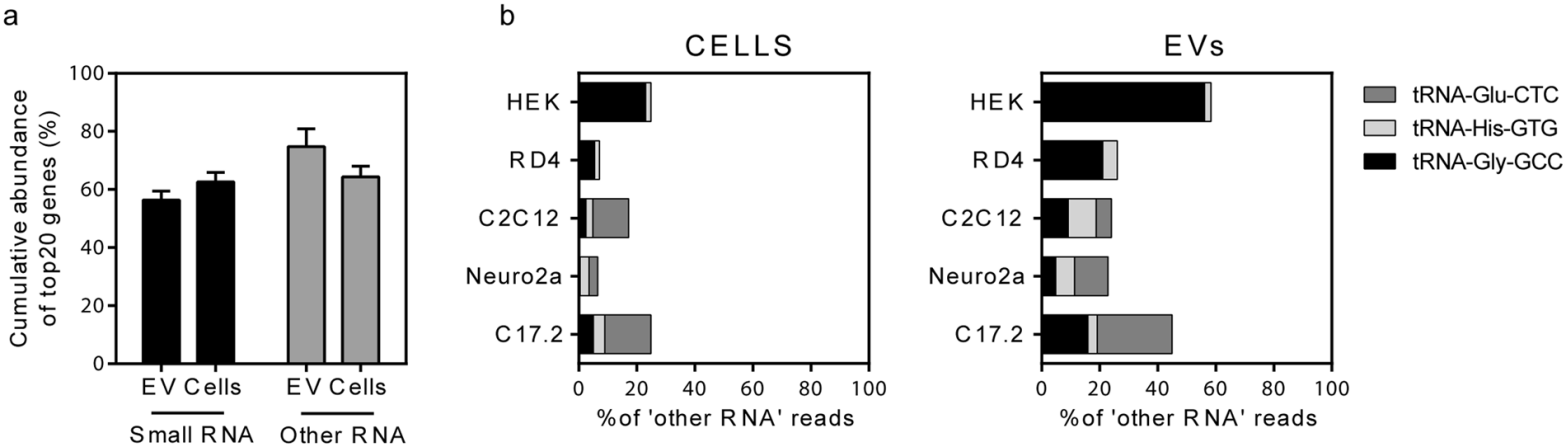
Contribution of top ranking genes within ‘small RNA’ and ‘other RNA’ categories. (**a)** A low number of highly abundant transcripts were found to contribute to the overall sample heterogeneity both in cells and EVs, being more pronounced for the ‘other RNA’ as opposed to ‘small RNA’ category. **(b)** The vesicular ‘other RNA’ category was especially rich in fragments originating from tRNAs carrying Glu-CTC, His-GTG and Gly-GCC anticodons (right panel).

miRNA expression scatter plots unfolded a good correlation between miRNA levels in EVs and their parental cells (Supplementary Figure S3). This was further confirmed by hierarchical clustering analysis of miRNA signatures, where the vesicular miRNA patterns resembled their parental cells more closely than other EV types throughout our entire dataset. Nevertheless, a handful of individual miRNA sequences deviated from this relationship. For example, miR-451a, miR-144, miR-6087 and miR-451a, miR-6239, miR-6240, miR-6236 in human and mouse samples, respectively were found at higher than expected levels in EVs as compared to the cellular background. Nevertheless, potential contamination with bovine serum derived miRNAs was observed to a low extent (Supplementary Table S2). The top 20 EV-associated miRNAs overlapped largely with the top 20 miRNA genes of the respective source cell, with some exceptions (Supplementary Table S3, grey-shaded entries). Of note, in addition to a unanimously identified miR-21, other miRNAs (miR-378a, miR-30d, miR-99b and mir-92a-1) appeared in the top 20 miRNA gene list in 4/5 of all studied EVs (Supplementary Table S3, underlined entries).

Inversely to the earlier observation with miRNAs, there was no clear correlation between piRNA read counts in EVs and cells, though we did detect a few piRNAs (e.g. hsa_piR_001356 (DQ571873) and mmu_piR_002962 (DQ548183)) which tended to accumulate in EVs (Supplementary Figure S4). Interestingly, mmu_piR_000935 (DQ541777) was the most abundant piRNA in almost all mouse samples (both cells and EVs, except for Neuro2a- and C2C12 EVs), whereas such uniformity of piRNA enrichment was not seen for human samples. When looking at the characteristic size range of mature piRNAs (27–35 nt) as well as their 5′-end bias for uracil (U), a limited number of reads were seen to comply with these criteria (Supplementary Figure S5). We also detected that some of the reads annotating to piRNA loci could potentially represent tRNAs. This, however was seen to have a low to negligible influence on the overall small RNA transcriptome with up to 1.8% and 0.09% of all the annotations suffering from piRNA/tRNA annotation ambiguity. Yet, we would like to emphasize that the current piRNA annotations rather denote reads mapping to piRNA loci than piRNAs *per se*.

#### EVs are rich in fragments derived from tRNA-, Y RNA- and protein coding genes

In addition to ‘small RNAs’ we decided to look into the specific composition of ‘rRNA’ and ‘other RNA’ categories, which represented the majority of the 17–35 nt long RNA transcriptome in EVs. The majority of rRNA sequences in cells were large and small subunit rRNA fragments with some contribution from mitochondrial rRNA (Supplementary Figure S6, upper panel). Generally, these rRNA patterns were similarly reflected in human-derived EVs. On the other hand, mouse-derived vesicles showed a higher proportion of vesicular small subunit ribosomal sequences. However, contrary to cell samples, mitochondrial rRNA fragments were practically absent in all EV samples (<0.2% of ‘rRNA’ reads).

Both in cells and EVs, the main RNA species within the ‘other RNA’ category were tRNAs, protein coding genes and miscellaneous RNA (incl. Y RNA) fragments (Supplementary Figure S6, lower panel). It should be noted that only a small number of individual genes contributed to the vast majority of total RNA reads (and thus to total bulk mass of RNA) both in the case of ‘small RNA’ and ‘other RNA’, as illustrated by the cumulative histograms of RNA reads (Supplementary Figure S7). Indeed, while the top 20 ‘small RNAs’ and ‘other RNAs’ in cells covered an average of ~60% of total RNA in the respective categories, the top 20 ranking annotations of vesicular ‘other RNA’ covered as much as 75% of the total ‘other RNA’ amount (Fig. 4A). This illustrates the abundance of the top ranking ‘other RNA’ genes, mostly annotating to tRNA and Y RNA loci. Interestingly, vesicular tRNA fragments were mostly 30–34 nt long and derived from tRNA-Gly-GCC, tRNA-His-GTG and tRNA-Glu-CTC genes, with a total contribution of 23%-58% of total ‘other RNA’ reads throughout the tested EVs (Fig. 4B, Supplementary Figure S8). The majority of Y RNA annotations for mouse originated from Rny1 and for human, from Y_RNA (ENSG00000201778), RNY1, RNY4 and RNY5 genes. As the human genome also carries many Y RNA pseudogenes, a considerable number of reads were annotated also to RNY4P10 and RNY4P17 pseudogenes. Similarly to earlier reports^22,30^, a majority of human Y RNA fragments were 30–34 bases in length whereas the length of mouse Y RNA sequences peaked at 28 nucleotides (Supplementary Figure S8).

Overall, the abovementioned observations clearly demonstrate that individual miRNA species represented only a relatively small fraction of all short 17–35 nt RNA sequences in EV samples. On the other hand, other RNA species (e.g. rRNA fragments and specific tRNA fragments) were more abundant in all tested cell line EVs. This could also be seen from the list of overall top-ranking RNA genes across all RNA categories (‘small RNA’, ‘rRNA’ and ‘other RNA) in this study. While in the cell samples the prominence of individual miRNAs was very clear, the EV samples were dominated by highly abundant ‘rRNA’ and ‘other RNA’ annotations (Supplementary Table S4). However, there were noticeable and particularly important dissimilarities between different cell type-derived EVs, e.g. HEK EVs were particularly rich of rRNA fragments whereas C17.2 and C2C12 EVs contained the largest proportion of ‘small RNA’ (and thus also miRNA) sequences (Supplementary Figure S9).

### RNA binding proteins in the EV proteome

Next, we set out to explore whether the EV proteome, particularly the repertoire of RNA-binding proteins would correspond and contribute to our observations on the types of RNA species in EVs. For this, we focused on EVs derived from HEK and C2C12 cells given their disparate small RNA content, as highlighted above.

**Figure 5:**
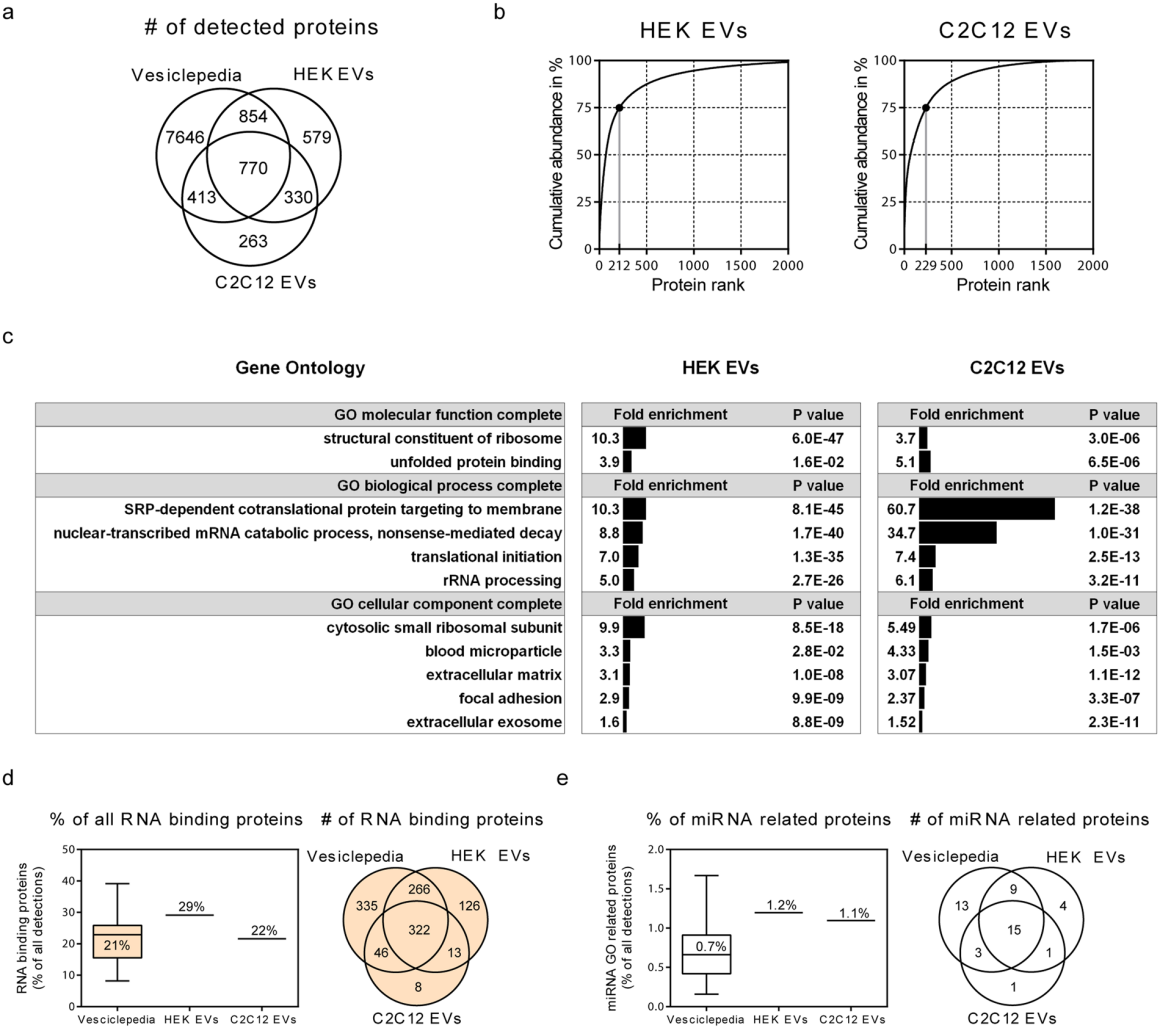
Proteomic analysis of HEK- and C2C12 EVs. **(a)** Venn diagram showing protein identifications in HEK- and C2C12 EVs compared to Vesiclepedia database. **(b)** Cumulative abundance of proteins in EVs (sorted by abundance/MS Area rank). Both proteomes showed a low number of highly abundant proteins accounting for 75% of the total protein mass. **(c)** Gene Ontology (GO) enrichment analysis of all proteins revealed an overrepresentation of GO classes related to protein targeting to membrane and mRNA catabolic processes (Panther Statistical Overrepresentation Test, full information in Supplementary Figures S10 and S11). The reference protein list that was used to calculate fold enrichment included all protein identifications of the respective EV sample. **(d**,**e)** Fraction of proteins from the GO terms ‘RNA binding’ proteins (**d**) and ‘miRNA related’ (**e**) (full list of GO terms in Supplementary Table S8) in HEK and C2C12 EVs compared to Vesiclepedia database. Vesiclepedia database was limited to experiments with 500 or more protein identifications.

In both HEK and C2C12 EVs ~2000 proteins were identified, of which most (64% and 67%, respectively) overlapped with annotations in the manually curated Vesiclepedia database^31^. However, we also detected a number of proteins that were not annotated in Vesiclepedia (Fig. 5A). The 212 and 229 most abundant EV proteins (ranked according to MS Area) accounted cumulatively for 75% of the total amount of protein in HEK and C2C12 samples, respectively (Fig. 5B). ~90% of total protein mass in both HEK and C2C12 was found to be covered by the top 500 most abundant EV proteins. In addition to numerous RNA binding proteins, these proteins included typical EV markers (CD81, CD9, PDCD6IP, MFGE8 and SDCBP) as well as RAB proteins that are involved in EV biogenesis^1^.

To investigate the characteristics of the conservative set of proteins that make up the bulk (75%) of the EV proteome, we employed the Panther Statistical Overrepresentation Test^32^ using a reference list of all detected protein identifications per EV source. Among the commonly overrepresented Gene Ontology (GO) classes for both vesicle types we found overrepresentation of entries related to ribosomes, mRNA catabolism, translation initiation, protein targeting to membranes, unfolded protein binding, extracellular matrix, focal adhesion, blood microparticles and extracellular exosomes (Fig. 5C). However, there were some differences between HEK EVs and C2C12 EVs as well. Of note, in the top 75% of the protein mass of C2C12 EVs we also identified overrepresented GO classes related to signalling (e.g. TNF, NIK/NF-κB, Wnt and MAPK pathways), cell cycle regulation, and ER and Golgi membrane components, whereas these classes were not significantly overrepresented in HEK EVs (Supplementary Figures S10 and S11). However, when performing the Panther Statistical Enrichment Analysis (i.e. an analysis that takes into account the expression level of individual proteins) as opposed to the Overrepresentation Test (i.e. the test only accounts for detection of individual proteins irrespective of their expression level), highlighted GOs included Wnt signalling, threonine-type peptidase activity, ncRNA processing, RNA stability regulation, together with other rRNA- and mRNA-related gene ontologies (Supplementary Tables S5 and S6).

As we were mainly interested in correlating this proteomics data to our small RNA sequencing data, we next, we focused on the RNA binding proteins in the datasets. For this, we identified all proteins in HEK and C2C12 EV samples that were classified as ‘RNA binding’ (GO:0003723) according to the QuickGO database (http://www.ebi.ac.uk/QuickGO/). This set of proteins constituted 29% and 22% of all identified proteins in HEK and C2C12 EV samples, respectively, which is in line with the mean reported frequency (21%) of RNA binding proteins in Vesiclepedia database (Fig. 5D).

We next performed a similar analysis for miRNA-related proteins by first identifying proteins from a list of 50 miRNA related GO terms, i.e. a custom GO slim category ‘miRNA related’ (Supplementary Table S8) and thereafter downloading the corresponding protein annotations using the QuickGO browser. Both the EVs as well as Vesiclepedia datasets were found to contain ~1% proteins annotated as miRNA related (Fig. 5E, Supplementary Table S8). Additionally, most ‘miRNA related’ proteins in our dataset were expressed at very low levels, with a mean rank number of ~1200 and contribution to the total EV protein mass by ~0.9% for both samples (Supplementary Tables S9 and S10). Exceptions included EIF4E, NSUN2, METTL3 and RAN that ranked within the top 500 most abundant proteins (representing cumulatively 90% of total EV protein content). Further analysis of HEK EV- and parental cell proteomes revealed that in cells, the abundance distribution pattern of ‘RNA binding’ proteins and ‘miRNA related’ proteins was similar (Supplementary Figure S12). Yet, the ‘miRNA related’ proteins in HEK EVs were more likely to have a low expression level (as compared to the abundance distribution of ‘RNA binding’ proteins) and their total amount was nearly two times lower than in HEK cells. Altogether, confirming the low relative contribution of ‘miRNA related’ proteins to the EV proteome.

**Figure 6:**
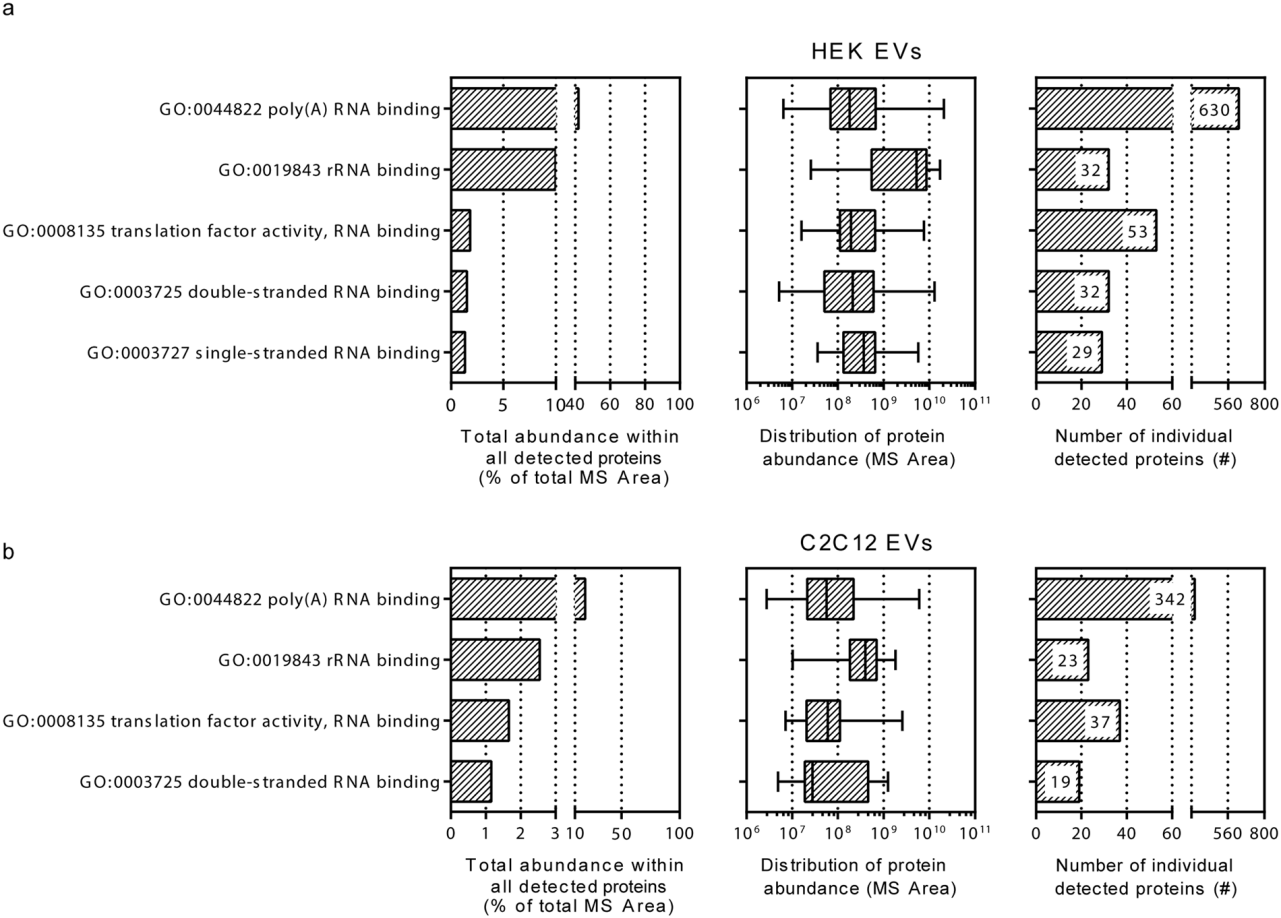
Gene Ontology (GO) term based analysis of RNA binding proteins in HEK- and C2C12 EVs. The RNA binding proteins (GO:0003723) were categorized under the respective child terms and analyzed in terms of their number and abundance ranking. Proteins constituting >1% of total protein MS Area are depicted. GO terms with <1% contribution to the proteome can be found in Supplementary Figure S13.

In order to further understand which RNA-binding protein classes are represented in the data sets and how these data sets correspond to the small RNA sequencing results, we manually curated the identified proteins according to their respective child terms within the GO class 0003723 ‘RNA binding’. Proteins were ranked according to their total abundance (total MS Area) and the number as well as abundance distribution of these proteins within each GO child term was verified (Fig. 6A,B, Supplementary Figure S13). GO classes contributing most to the total EV proteome for HEK as well as C2C12 included terms describing poly(A)- and rRNA binding proteins, double- and single-stranded RNA binding proteins as well as translation-related GO subclasses (Supplementary Table S11). It should be noted that protein evidence within individual GO terms is not mutually exclusive as the presence of one given protein may be reflected in several of the GO child terms (i.e. a protein can be both rRNA binding, mRNA binding and have translation factor activity).

## Discussion

EVs have gained widespread interest due to their native components such as proteins and RNA, thereby serving as a rich source of molecules with therapeutic and biomarker potential. EV-mediated transfer of bioactive components, as well as their involvement in disease pathogenesis has been described in numerous studies. However, a lack of detailed knowledge of the cooperation of biomolecules with basic biological mechanisms still impedes employing the full potential of EVs for therapeutic interventions.

This study involves small RNA transcriptomics and proteomic approach to describe the inherent heterogeneity of EV cargo. Even though several EV (small) RNA profiling studies have previously been published^22,24,25,29,33–35^, many of these concentrate on only one cell type. Hence, there is a strong need for additional comprehensive comparisons with improved capacity to unfold biological mechanisms controlling EV cargo loading.

The study at hand investigated the overall RNA diversity of parental cells and EVs from five different cell types. Expectedly, the parental cells predominantly contained ‘small RNAs’, of which a great majority represented miRNA sequences, strongly affirming the reliability of the applied methodology. In contrast, the ‘small RNA’ content in EVs was modest and highly variable across the studied samples. Nevertheless, there was a large overlap and a good correlation of vesicular and parental cell content in terms of the relative abundance of miRNAs. Yet, this correlation excluded certain individual miRNAs, e.g. miR-451a which was more enriched in EVs than would be expected from the parental cell background. Enrichment of this non-canonically processed miR-451a has been reported in a number of studies^16,18,35–37^ and as a highly abundant miRNA in blood^38^, its enrichment in FBS and thereby carryover to cell-culture settings could lead to misinterpretation of the biological abundance^39^. The usage of serum supplemented medium prior to conditioned media collection may also have had an influence in our dataset, revealing potential serum-confounding miRNAs with read counts in vesicular samples exceeding those of parental cells. Screening the top three FBS-enriched miRNAs previously reported by Wei *et al*.^39^ (in addition to miR-451a), we indeed found potential FBS-confounding miRNAs, as they were less abundant in cells than EVs. Nevertheless, this observation was either not consistent throughout the tested cell lines or the particular miRNAs were low abundant by not ranking among the top 50 most abundant miRNAs (covering 84–92% of the miRNome in cells and EVs). Hence, when focusing on total EV miRNA levels, the concern of miRNA carryover from FBS into our EV samples is low.

In addition to miRNAs, vesicle samples throughout the tested cell lines exhibited a remarkable richness in sequences derived from piRNA loci, which however lacked the correlation with their parental cell expression level. The vesicular enrichment of piRNAs was quite different from miRNAs and mostly showed cell-line specific EV enrichment of certain annotations. Yet, piR_000935 stood out as the most abundant piRNA in practically all mouse cells and EVs and thereby caught our attention. As the genomic location of piR_000935 overlaps with Rny1 gene (Ro-associated Y1), we cannot rule out that the sequences at hand might instead represent 5′ Y RNA fragments, which due to the choice of stepwise annotation could have become underrepresented (Y RNAs are classified under ‘other RNA’ category, ‘miscellaneous RNA’ biotype).

Whereas ‘small RNA’ sequences in EVs represented a relatively modest part (22%) of all annotated reads, we found a large amount (60%) of rRNA fragments within the 17–35 nt long sequences. Overall, the rRNA content in EVs reflected the pattern of their parental cells. Interestingly, in human samples, the majority of these fragments were derived from the large ribosomal subunit sequences, whereas in mouse samples we detected a higher quantity of small subunit fragments. Additionally, though mitochondrial rRNA fragments were detected in low amounts in cell samples, virtually none of these fragments was found in EV samples. Despite performing stringent evaluation on cell viability throughout EV production, we cannot rule out that some of the rRNA in our EV samples is derived from apoptotic bodies. Yet, in EVs as compared to cells, the overall level of rRNA fragments is too high to merely be attributed to contaminating factors. In fact, even though contradicting evidence on the vesicular rRNA content exist, the vast majority of vesicular deep sequencing studies report a strong predominance of rRNA fragmentation products in EVs^22,40–42^, corroborating well with the data presented here. The presence of rRNA fragments have often been denoted as impurities in EV preparations or unspecific degradation products, displaying a high abundance that can already be anticipated from their richness in the cell. Yet, an increasing number of studies have highlighted the precise cleavage patterns of rRNA^42,43^. These fragments (when overexpressed) are able to affect cell viability^44^ and are capable of entering the RNA interference pathway^40,45^, indicating a potential role in gene regulation in recipient cells, even across kingdoms^46^.

The most prominent biotypes in the ‘other RNA’ category were tRNAs-, protein coding- and miscellaneous RNA (including Y RNAs). Regardless of the number of detected distinct RNAs, the bulk of the ‘other RNA’ reads originated from around 20 transcripts, with tRNAs and Y RNAs being the most abundant RNA species. The high abundance of tRNAs as well as enrichment of particular tRNA/Y RNA-derived fragments have previously been reported in several EV-based studies, despite the employment of short read-length sequencing^22,24,25,47^. Herein, we found a notable predominance of vesicular tRNA-Gly-GCC, tRNA-Glu-CTC and tRNA-His-GTG corroborating well with previous findings^6,25^. Since we restricted the analysis to reads of 17–35 nucleotides in length, the observed sequences likely correspond to tRNA fragmentation products, with a predominance of 28–35 nucleotides long sequences of uniquely tRNA-annotated reads for both cellular as well as vesicular samples. To date, a wide variety of tRNA derived fragments (termed sitRNAs, tiRNA, tRNA halves, tRNA fragments and tsRNAs etc.) have been identified, some with a direct capability to inhibit protein synthesis and function as miRNAs (reviewed in 88). It has yet to be determined if any of the detected fragments in the current dataset could belong to any one of these subclasses and contribute to any biological functions.

The highly variable and relatively scarce representation of miRNAs in EVs led us to further investigate the association of the small RNA transcriptome to the vesicular proteome by choosing HEK and C2C12 EVs as a basis for our further analysis. Similarly to transcriptomic results in which a relatively small number of RNA sequences contributed to a large proportion of total detected RNA reads, ~200 most abundant proteins were found to make up 75% of the total protein mass in EVs. Also, in line with the finding of a high abundance of ribosomal, coding and tRNA fragments in the sequencing data set, we discovered high levels of rRNA-, poly(A)- and tRNA binding proteins in the proteomic dataset for both HEK- and C2C12 EVs.

As one of interests of the study was to verify whether the RNA repertoire of the EVs would correspond to the proteome, we were especially interested in the correlation of RNA binding proteins with respect to the related RNA species. Since passive as well as active mechanisms of miRNA loading into EVs have been suggested^48–52^, we first decided to check the interplay of miRNAs and their binding proteins. Though C2C12 EVs were substantially richer in miRNAs than HEK EVs, we were unable to detect any major differences in the miRNA-related proteome. Based on our custom-curated GO list we detected 33 miRNA-related proteins, showing a negligible contribution (<1%) to the proteome for both samples. From the RISC-loading complex, only a very low amount of DICER1 and TARBP1 (found only in HEK EVs) as well as a low amount of MOV10 RISC complex RNA helicase (in both samples) were detected. Additionally, in contrast to previous studies^53,54^, we were unable to detect any Argonaute proteins. Considering the discrepant abundance of miRNAs in these EV samples together with a good correlation of cellular and vesicular miRNA levels, regarding this dataset, it is difficult to speculate whether miRNA sorting into EVs is dependent on a specific miRNA binding protein. Number of different proteins have been demonstrated to be involved in miRNA secretion within EVs, thus multiple sorting mechanisms may co-exist simultaneously.

In contrast to the relative depletion of miRNA reads, EVs showed a high number of piRNA sequences. Initially, we hypothesized that this is contributed by the EV protein composition. However, this was not the case as none of the proteins annotated as ‘piRNA binding’ in the GO database were found in EVs. The presence of piRNA binding proteins in EVs has so far (in low amounts) been reported only in a few studies^55–57^. Herein, despite the high number of piRNA sequences, piRNA-related proteins remained undetected, aligning well with our observation that the sequences annotated as piRNAs in this study rather represent reads mapping to piRNA loci.

A high abundance of rRNA reads in all EV samples led us to investigate the ‘rRNA binding’ (GO:0019843) proteins, which were found to constitute ~10% and ~3% of HEK and C2C12 EV proteomes, respectively. Considering the richness of reads annotating to rRNA loci, it seems that the rRNA is largely disconnected from the loading of ribosomal proteins. Concomitantly, we cannot exclude that the high abundance of rRNA reads could be brought about due to limitations in EV sample preparation, though contamination markers (e.g. calnexin) in vesicle preparations were present in negligible amounts.

The highest number and contribution to the proteome for both datasets was seen for proteins classified under “poly(A) RNA binding” term. Though, a few protein coding genes did rank high in the EV transcriptome, we believe that the short read-length sequencing used in the present study is not sufficient to serve as a basis for further elaborations. The high contribution of proteins described by “translation factor activity” term was found to corroborate well with the increased abundance of tRNA sequences in the EV secretome, thereby supporting earlier studies describing the abundance of transcriptional/translational factors in the EV proteome and transcriptome^29,40,58^.

An increasing number of studies are concentrating on understanding the loading and interplay of bioactive EV macromolecules. This study, combining the vesicular RNA and protein cargo composition, serves as a valuable source of information not only in terms of knowledge in basic EV biology but also for the development of EV-based carrier systems for therapeutic RNA interventions. Nonetheless, as we are only considering a handful of cell types, there is still a need for additional studies utilizing other EV enrichment methods and cell types to fully understand and seize the underlying potential of vesicular biology for therapeutics.

## Methods

### EV purification

Neuro2a, RD4, C17.2, C2C12 and HEK293T cell-lines were maintained in high-glucose (4.5 g/l) Dulbecco’s modified Eagle’s medium with GlutaMax (DMEM GlutaMax, Thermo Fisher Scientific, Waltham, MA, USA) supplemented with either 10% (Neuro2A, RD4, C17.2 and HEK293T) or 20% (C2C12) fetal bovine serum (FBS, Thermo Fisher Scientific, Waltham, MA, USA). All cells were cultured in a humidified atmosphere at 37 °C and 5% CO_2_. 3 × 10^6^ (C2C12, Neuro2a, RD4, C17.2) or 5 × 10^6^ (HEK293T) cells were first seeded onto 15 cm cell culture dishes. After 24 hours, cells were washed with 0.01 M phosphate buffered saline (PBS) to ensure removal of any residual FBS before a media change to OptiMEM (Thermo Fisher Scientific, Waltham, MA, USA) supplemented with 1% Antibiotic Antimycotic Solution (Sigma-Aldrich, Saint-Louis, MO, USA). After 48 hours, cell viability was evaluated by trypan blue exclusion method and cultures displaying <95% viable cells were excluded from further processing. The supernatant from the cell cultures passing the quality control step was collected and EVs were harvested by differential ultracentrifugation (UC). Briefly, conditioned medium (CM) was spun at 300 g for 5 minutes, followed by a spin at 1,500 g for 10 minutes and 0.22 µm filtration to remove dead cells, cell debris and larger particles. EV pelleting was performed by two consecutive pelleting steps at 110,000 g for 90 minutes, with an intermediate washing of the EV pellet in 25 ml of PBS. The final EV pellet was resuspended in PBS and either stored at −80 °C for proteomic analysis as indicated below or mixed with Trizol LS (Thermo Fisher Scientific, Waltham, MA, USA) and stored at −80 °C until RNA extraction for small RNA sequencing analysis. All UC steps were performed at 4 °C, using a fixed angle Type 70 Ti rotor from Beckman Coulter (Beckman Coulter, Inc., Brea, CA, USA).

### EV characterization

Nanoparticle Tracking Analysis (NTA) was performed by using the NanoSight NS500 nanoparticle analyser (Malvern Instruments, Malvern, UK) to measure the size distribution and concentration of the purified EVs. The EV samples were diluted in PBS and the movement of the particles was recorded within five 30 seconds videos that were subsequently analysed using the NTA Software 2.3 (Malvern Instruments, Malvern, UK) at detection threshold 5, camera gain 350 and shutter setting 800.

Transmission electron microscopy (TEM) was performed by applying the purified HEK293T EV sample onto Formvar/Carbon type B coated electron microscopy grid (Ted Pella Inc., Redding, CA, USA). The grid was washed with double distilled H_2_O, blotted dry with filter paper and stained with 2% uranyl acetate (Sigma-Aldrich, Saint-Louis, MO, USA) diluted in distilled water. The grid was dried with filter paper and analysed with a FEI Tecnai 10 transmission electron microscope (FEI) at an accelerating voltage of 100 kV.

Western blotting (WB) was performed as described previously^59^. Molecular weight was estimated according to PageRuler Plus Prestained Protein Ladder (10 to 250 kDa; Thermo Fisher Scientific, Waltham, MA, USA). For probing, 1:1000 primary antibody dilutions were used for anti-ALIX [ab117600, Abcam], anti-TSG101 [ab30871, Abcam], anti-Calnexin [ab22595, Abcam], anti-SDCBP (syntenin) [TA504796, clone OTI2H6, OriGene] and 1:5000 dilution anti-ß-actin [A5441, clone AC-15, Sigma-Aldrich]. For detection, 1:15,000 dilution of secondary antibodies (goat anti-mouse IRDye 800CW to detect ALIX and ß-actin; goat anti-mouse IRDye 680LT to detect SDCBP; goat anti-rabbit IRDye 800CW to detect Calnexin and goat anti-rabbit IRDye 680LT to detect TSG101; all from LI-COR Biosciences, NE, USA) were employed. The membranes were visualized on the Odyssey infrared imaging system and further analysed by using Image Studio Lite Version 5.2 (both from LI-COR Biosciences, NE, USA).

### Sample preparation for small RNA sequencing

Total RNA extractions were performed according to manufacturer’s instructions using TRIzol and TRIzol LS (both Thermo Fisher Scientific, Waltham, MA, USA) for cells and EVs respectively. Improved RNA precipitation was gained by adding 2 µl of PolyAcryl Carrier PC 152 polymer (Molecular Research Center, Inc., Cincinnati, OH, USA) per reaction. The RNA integrity of the cell samples was verified on Bioanalyzer RNA 6000 Pico Total RNA Kit (both Agilent Technologies, Lanarkshire, UK) and the RNA concentration for all samples was measured with the Qubit 2.0 Flurometer by using the Qubit RNA HS Assay Kit (Thermo Fisher Scientific, Waltham, MA, USA). 250 ng of total RNA was subjected to small RNA library preparation by using the NEBNext Multiplex Small RNA Library Prep Set 1 for Illumina (NEB, Ipswich, MA, USA) kit according to the manufacturer’s instructions. The barcoded samples were size selected on the 6% Novex TBE PAGE gel (Thermo Fisher Scientific, Waltham, MA, USA) and the fragments corresponding to microRNA range were cut and subjected to purification with the NucleoSpin Gel and PCR Clean-up kit (Macherey-Nagel, Düren, Germany). Thereafter, the products were quantified by using the KAPA Library Quantification Kit (Cat.No.KK4824, Kapa Biosystems, London, UK) and pooled at equimolar ratio. Two libraries (technical replicates) were generated in parallel, each one eventually containing a pool of 12 barcoded samples. The readymade libraries were further checked on the High Sensitivity D1000 ScreenTape (Agilent Technologies, Lanarkshire, UK) and quantified by using the KAPA Library Quantification Kit to enable precise loading of the flow cell. The clusters were generated by using the cBot and one replicate per lane was sequenced on the HiSeq 2500 (HiSeq Control Software 2.0.12.0/RTA 1.17.21.3, Illumina Inc., San Diego, CA, USA) with a 1 × 51 setup in RapidRun mode.

### Sample preparation for liquid chromatography tandem mass spectrometry (LC-MS/MS)

UC-purified EVs and HEK cells were analysed by LC-MS/MS as described previously^28^. The Gene Ontology (GO) term enrichment and -overrepresentation analysis were performed by using the Protein ANalysis THrough Evolutionary Relationships (PANTHER) software (release 20161020)^32^. The abundance of distinct proteins was calculated from the MS Area of the particular protein over the total MS Area of the sample proteome. The Panther Statistical Overrepresentation Test was based on reference protein list containing all protein identifications of the input list.

The fraction of RNA binding proteins was calculated by basing on the list of “RNA binding” proteins (GO:0003723) obtained via the QuickGO browser (http://www.ebi.ac.uk/QuickGO/, Gene Ontology Database release 2016-10-27) as well as human/mouse proteomic entries in the Vesiclepedia^31^ database for experiments with a minimum of 500 proteins identified. For GO term analysis of miRNA related proteins, a list of 50 miRNA related GO terms was compiled, followed by downloading the corresponding protein annotations using the QuickGO browser. All proteomic analysis is based on unique protein identifiers, thereby taking into account different isoforms of the same protein.

### Small RNA sequencing data analysis

Raw sequencing reads were quality controlled by FastQC^60^ analysis and subjected to adapter removal by Cutadapt/1.9.1^61^. All reads with an adapter and a length of 17–35 bases (filtering with BBMap release 35.40^62^) were subjected to subsequent mapping on the Ensembl 38.85 releases of the mouse and the human genome by using Bowtie1 (release 0.12.6)^63^ in -v1 alignment mode and best alignment stratum reporting option.

Annotation was performed in a stepwise manner with HTSeq (release 0.6.1)^64^ in stranded mode. Briefly, reads were first annotated against ‘small RNAs’, followed by ‘ribosomal RNA’ and ‘other RNA’ annotations with intermediate filtering steps allowing only the reads with no found feature to continue down the pipeline. Gene biotype classification was done following the classification details in Vega Genome Browser release 68. MicroRNA annotations were retrieved from the miRBase^65^ release 21, snoRNA and snRNA annotation from Ensembl 38.85^66^ annotation file and piRNAs from piRNAbank^67^ followed by the UCSC liftOver^68^ of the hg18 and mm8 chromosomal coordinates to genome release 38 coordinate system. The ribosomal RNA annotations consisted of mitochondrial rRNA annotations from Ensembl 38.85 and a combination of the UCSC Table Browser hg38/mm10 RepeatMasker ribosomal RNA entries with ribosomal RNA annotations from the Ensembl 38.85 release that using BEDTools/2.26.0^69^ had less than 50% reciprocal overlap with the aforementioned RepeatMasker entries. The ‘other RNA’ annotation gathered annotation entries from Ensembl 38.85 which were not included in either of the ‘small RNA’ or ‘rRNA’ annotation files, plus tRNA annotations of the respective genomes which were retrieved from UCSC Table Browser. The absence of human RNY5 and its pseudogenes’ annotations from Ensembl 38.85 was compensated by including manual annotation entries from Ensembl GRCh38.p10 to the gained ‘other RNA’ annotation file. tRNA read counts originating from different loci were pooled according to the isoacceptors of a given tRNA. If applicable, the miRNAs/piRNAs with read counts ≥3 were normalised over the total number of miRNA/piRNA reads in the respective samples. The online analysis software Morpheus (available from the Broad Institute; https://software.broadinstitute.org/morpheus) was used to generate heatmaps as well as perform hierarchical clustering. For both analysis, normalised read counts were used and entries with zero read count throughout the tested samples were excluded. Hierarchical clustering was performed by utilizing one minus Pearson coefficient as the clustering distance measure. Base composition analysis for reads mapping to piRNA loci was performed with FastQC analysis followed by aggregating the results with MultiQC v1.3^70^.

### Statistical Analysis

Statistical analyses were performed using GraphPad Prism Version 6 (GraphPad Software, Inc., La Jolla, CA, USA).

### Data availability

The small RNA sequencing data has been deposited to NCBI Sequence Read Archives under accession number SRP118970 (BioProject: PRJNA408072).

## Electronic supplementary material

Supplementary Information

## References

[1] Colombo M, Raposo G, Théry C (2014). Biogenesis, Secretion, and Intercellular Interactions of Exosomes and Other Extracellular Vesicles. Annu. Rev. Cell Dev. Biol..

[2] Muralidharan-Chari V (2009). ARF6-Regulated Shedding of Tumor Cell-Derived Plasma Membrane Microvesicles. Curr. Biol..

[3] Taylor RC, Cullen SP, Martin SJ (2008). Apoptosis: controlled demolition at the cellular level. Nat. Rev. Mol. Cell Biol..

[4] Akers JC, Gonda D, Kim R, Carter BS, Chen CC (2013). Biogenesis of extracellular vesicles (EV): Exosomes, microvesicles, retrovirus-like vesicles, and apoptotic bodies. Journal of Neuro-Oncology.

[5] Chen X, Liang H, Zhang J, Zen K, Zhang CY (2012). Secreted microRNAs: A new form of intercellular communication. Trends in Cell Biology.

[6] Baglio SR (2015). Human bone marrow- and adipose-mesenchymal stem cells secrete exosomes enriched in distinctive miRNA and tRNA species. Stem Cell Res. Ther..

[7] Squadrito MLL (2014). Endogenous RNAs Modulate MicroRNA Sorting to Exosomes and Transfer to Acceptor Cells. Cell Rep..

[8] Valadi H (2007). Exosome-mediated transfer of mRNAs and microRNAs is a novel mechanism of genetic exchange between cells. Nat. Cell Biol..

[9] Skog J (2008). Glioblastoma microvesicles transport RNA and proteins that promote tumour growth and provide diagnostic biomarkers. Nat. Cell Biol..

[10] Pegtel DM (2010). Functional delivery of viral miRNAs via exosomes. Proc. Natl. Acad. Sci..

[11] Kosaka N (2010). Secretory mechanisms and intercellular transfer of microRNAs in living cells. J. Biol. Chem..

[12] Zhang Y (2010). Secreted Monocytic miR-150 Enhances Targeted Endothelial Cell Migration. Mol. Cell.

[13] Coenen-Stass, A. M. L., Mäger, I. & Wood, M. J. A. Extracellular microRNAs in Membrane Vesicles and Non-vesicular Carriers. (2015). 10.1007/978-3-0348-0955-9_2 26608198

[14] Zheng, B. *et al*. Exosome-Mediated miR-155 Transfer from Smooth Muscle Cells to Endothelial Cells Induces Endothelial Injury and Promotes Atherosclerosis. *Mol. Ther*. (2017). 10.1016/j.ymthe.2017.03.031 PMC5475247 28408180

[15] Thomou T (2017). Adipose-derived circulating miRNAs regulate gene expression in other tissues. Nature.

[16] Van Der Vos KE (2016). Directly visualized glioblastoma-derived extracellular vesicles transfer RNA to microglia/macrophages in the brain. Neuro. Oncol..

[17] Balusu S (2016). Identification of a novel mechanism of blood–brain communication during peripheral inflammation via choroid plexus‐derived extracellular vesicles. EMBO Mol. Med..

[18] Phinney DG (2015). Mesenchymal stem cells use extracellular vesicles to outsource mitophagy and shuttle microRNAs. Nat. Commun..

[19] Vickers KC, Palmisano BT, Shoucri BM, Shamburek RD, Remaley AT (2011). MicroRNAs are Transported in Plasma and Delivered to Recipient Cells by High-Density Lipoproteins. Nat Cell Biol..

[20] Arroyo JD (2011). Argonaute2 complexes carry a population of circulating microRNAs independent of vesicles in human plasma. Proc. Natl. Acad. Sci. USA.

[21] Dhahbi JM (2013). 5′-YRNA fragments derived by processing of transcripts from specific YRNA genes and pseudogenes are abundant in human serum and plasma. Physiol. Genomics.

[22] Nolte-’t Hoen ENM (2012). Deep sequencing of RNA from immune cell-derived vesicles uncovers the selective incorporation of small non-coding RNA biotypes with potential regulatory functions. Nucleic Acids Res..

[23] Batagov AO, Kurochkin IV (2013). Exosomes secreted by human cells transport largely mRNA fragments that are enriched in the 3′-untranslated regions. Biol. Direct.

[24] Balkom, B. W. M. van *et al*. Quantitative and qualitative analysis of small RNAs in human endothelial cells and exosomes provides insights into localized RNA processing, degradation and sorting. *J*. *Extracell*. *Vesicles* **4** (2015). 10.3402/jev.v4.26760 PMC4450249 26027894

[25] Tosar JP (2015). ment of small RNA sorting into different extracellular fractions revealed by high-throughput sequencing of breast cell lines. Nucleic Acids Res..

[26] Chiasserini D (2014). Proteomic analysis of cerebrospinal fluid extracellular vesicles: A comprehensive dataset. J. Proteomics.

[27] Kossinova OA (2017). Cytosolic YB-1 and NSUN2 are the only proteins recognizing specific motifs present in mRNAs enriched in exosomes. BBA -Proteins Proteomics.

[28] Nordin JZ (2015). Ultrafiltration with size-exclusion liquid chromatography for high yield isolation of extracellular vesicles preserving intact biophysical and functional properties. Nanomedicine Nanotechnology, Biol. Med..

[29] Lässer C (2016). Two distinct extracellular RNA signatures released by a single cell type identified by microarray and next-generation sequencing. RNA Biol..

[30] Vojtech L (2014). Exosomes in human semen carry a distinctive repertoire of small non-coding RNAs with potential regulatory functions. Nucleic Acids Res..

[31] Kalra H (2012). Vesiclepedia: A Compendium for Extracellular Vesicles with Continuous Community Annotation. PLoS Biol..

[32] Mi H, Muruganujan A, Thomas PD (2013). PANTHER in 2013: modeling the evolution of gene function, and other gene attributes, in the context of phylogenetic trees. Nucleic Acids Res..

[33] Bellingham SA, Coleman BM, Hill AF (2012). Small RNA deep sequencing reveals a distinct miRNA signature released in exosomes from prion-infected neuronal cells. Nucleic Acids Res..

[34] Lunavat TR (2015). Small RNA deep sequencing discriminates subsets of extracellular vesicles released by melanoma cells–Evidence of unique microRNA cargos. RNA Biol..

[35] Guduric-Fuchs J (2012). Selective extracellular vesicle-mediated export of an overlapping set of microRNAs from multiple cell types. BMC Genomics.

[36] Li CC (2013). Glioma microvesicles carry selectively packaged coding and non-coding RNAs which alter gene expression in recipient cells. RNA Biol..

[37] Cheloufi S, Dos Santos CO, Chong MMW, Hannon GJ (2010). A dicer-independent miRNA biogenesis pathway that requires Ago catalysis. Nature.

[38] Landgraf P (2007). A Mammalian microRNA Expression Atlas Based on Small RNA Library Sequencing. Cell.

[39] Wei Z (2016). Fetal Bovine Serum RNA Interferes with the Cell Culture derived Extracellular RNA. Sci. Rep..

[40] Lefebvre FA (2016). Comparative transcriptomic analysis of human and Drosophila extracellular vesicles. Sci. Rep..

[41] Miranda KC (2014). Massively parallel sequencing of human urinary exosome/microvesicle RNA reveals a predominance of non-coding RNA. PLoS One.

[42] Fiskaa, T. *et al*. Distinct small RNA signatures in extracellular vesicles derived from breast cancer cell lines. *PLoS One* **11** (2016). 10.1371/journal.pone.0161824 PMC5006963 27579604

[43] Li Z (2012). Extensive terminal and asymmetric processing of small RNAs from rRNAs, snoRNAs, snRNAs, and tRNAs. Nucleic Acids Res..

[44] Hewson C, Capraro D, Burdach J, Whitaker N, Morris KV (2016). Extracellular vesicle associated long non-coding RNAs functionally enhance cell viability. Non-coding RNA Res..

[45] Wei H (2013). Profiling and Identification of Small rDNA-Derived RNAs and Their Potential Biological Functions. PLoS One.

[46] Weiberg A (2014). Fungal Small RNAs Suppress Plant Immunity by Hijacking Host. Science.

[47] Lambertz U (2015). Small RNAs derived from tRNAs and rRNAs are highly enriched in exosomes from both old and new world Leishmania providing evidence for conserved exosomal RNA Packaging. BMC Genomics.

[48] Shurtleff, M. J., Temoche-Diaz, M. M., Karfilis, K. V, Ri, S. & Schekman, R. Y-box protein 1 is required to sort microRNAs into exosomes in cells and in a cell-free reaction. *Elife* **5** (2016). 10.7554/eLife.19276 PMC5047747 27559612

[49] Villarroya-Beltri C (2013). Sumoylated hnRNPA2B1 controls the sorting of miRNAs into exosomes through binding to specific motifs. Nat. Commun..

[50] Koppers-Lalic D (2014). Nontemplated nucleotide additions distinguish the small RNA composition in cells from exosomes. Cell Rep..

[51] Janas T, Janas T, Yarus M (2006). Specific RNA binding to ordered phospholipid bilayers. Nucleic Acids Res..

[52] Teng Y (2017). MVP-mediated exosomal sorting of miR-193a promotes colon cancer progression. Nat. Commun..

[53] Melo SA (2014). Cancer Exosomes Perform Cell-Independent MicroRNA Biogenesis and Promote Tumorigenesis. Cancer Cell.

[54] Mantel P-Y (2016). Infected erythrocyte-derived extracellular vesicles alter vascular function via regulatory Ago2-miRNA complexes in malaria. Nat. Commun..

[55] Wang Z, Hill S, Luther JM, Hachey DL, Schey KL (2012). Proteomic analysis of urine exosomes by multidimensional protein identification technology (MudPIT). Proteomics.

[56] van Herwijnen MJC (2016). Comprehensive Proteomic Analysis of Human Milk-derived Extracellular Vesicles Unveils a Novel Functional Proteome Distinct from Other Milk Components. Mol. Cell. Proteomics.

[57] Fraser KB (2013). LRRK2 secretion in exosomes is regulated by 14-3-3. Hum. Mol. Genet..

[58] Stik G (2017). Extracellular vesicles of stromal origin target and support hematopoietic stem and progenitor cells. J. Cell Biol..

[59] Corso G (2017). Reproducible and scalable purification of extracellular vesicles using combined bind-elute and size exclusion chromatography. Sci. Rep..

[60] Andrews, S. FastQC: a quality control tool for high throughput sequence data. Available at: http://www.bioinformatics.babraham.ac.uk/projects/fastqc (2010).

[61] Martin M (2011). Cutadapt removes adapter sequences from high-throughput sequencing reads. EMBnet.journal.

[62] Bushnell, B. BBMap short read aligner. Available at: http://sourceforge.net/projects/bbmap/ (2016).

[63] Langmead B, Trapnell C, Pop M, Salzberg S (2009). Ultrafast and memory-efficient alignment of short DNA sequences to the human genome. Genome Biol..

[64] Anders S, Pyl PT, Huber W (2015). HTSeq-A Python framework to work with high-throughput sequencing data. Bioinformatics.

[65] Kozomara A, Griffiths-Jones S (2011). MiRBase: Integrating microRNA annotation and deep-sequencing data. Nucleic Acids Res..

[66] Yates A (2016). Ensembl 2016. Nucleic Acids Res..

[67] Sai lakshmi S, Agrawal S (2008). piRNABank: A web resource on classified and clustered Piwi-interacting RNAs. Nucleic Acids Res..

[68] James Kent W (2002). The human genome browser at UCSC. Genome Res..

[69] Quinlan AR, Hall IM (2010). BEDTools: A flexible suite of utilities for comparing genomic features. Bioinformatics.

[70] Ewels P, Magnusson M, Lundin S, Käller M (2016). MultiQC: Summarize analysis results for multiple tools and samples in a single report. Bioinformatics.

