# Supplementary material for: Heterogeneity and interplay of the extracellular vesicle small RNA transcriptome and proteome
Source: Sci Rep. 2018 Jul 17;8:10813. doi: 10.1038/s41598-018-28485-9 (PMC6050237; doi:10.1038/s41598-018-28485-9)
Supplement: Supplementary file 1 — Supplementary Information [file 41598_2018_28485_MOESM1_ESM.pdf]

Supplementary Information

Heterogeneity and interplay of the extracellular vesicle small RNA transcriptome and proteome

Helena Sork, Giulia Corso, Kaarel Krjutskov, Henrik J. Johansson, Joel Z. Nordin, Oscar P.B. Wiklander, Yi Xin Fiona Lee, Jakub Orzechowski Westholm, Janne Lehtiö, Matthew J.A. Wood, Imre Mäger, Samir EL Andaloussi

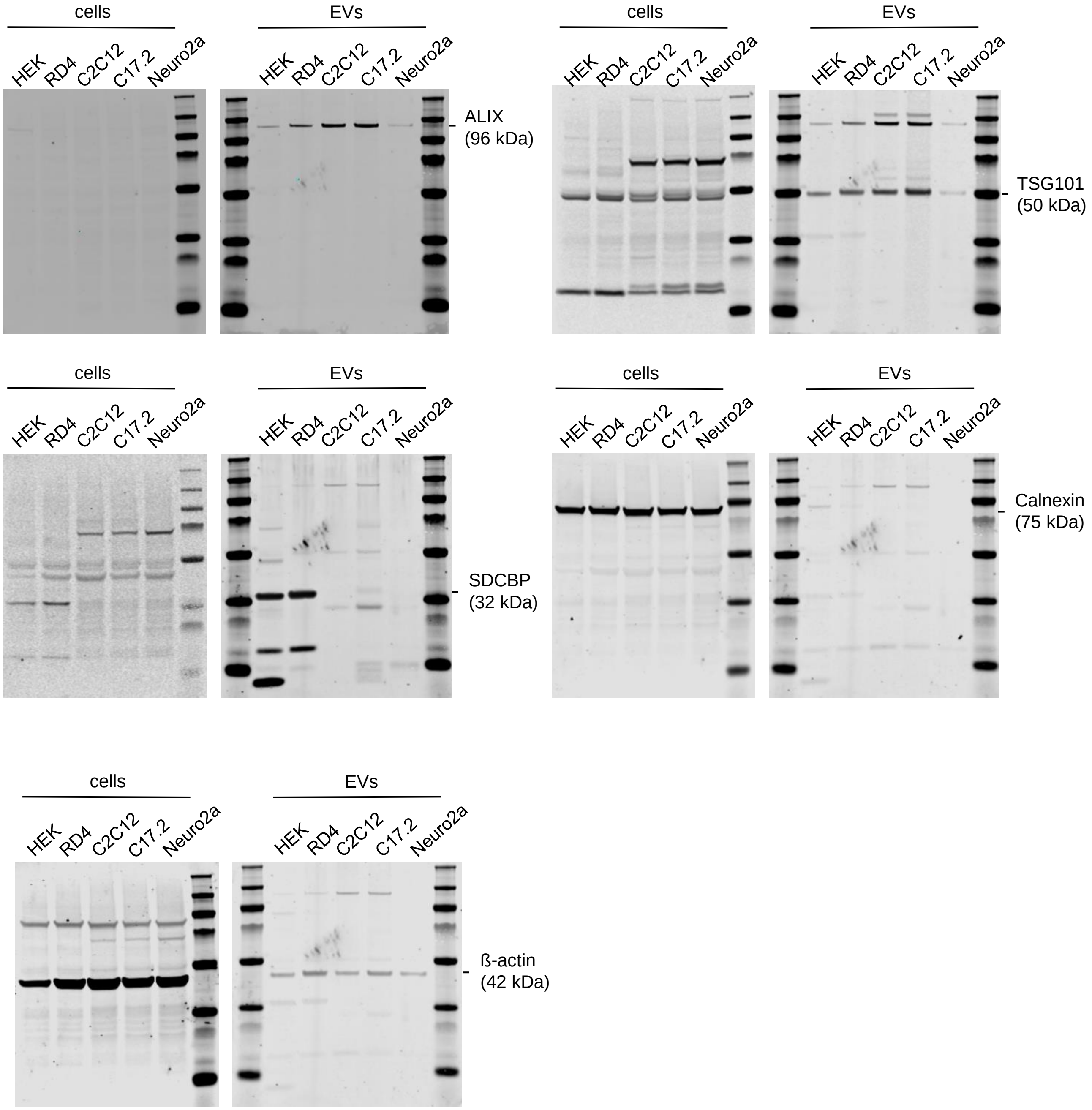

Supplementary Figure S1. Uncropped western blot images of cell- and EV samples. Cropped images can be found in Figure 1a. SDCBP (syntenin, human reactive antibody).

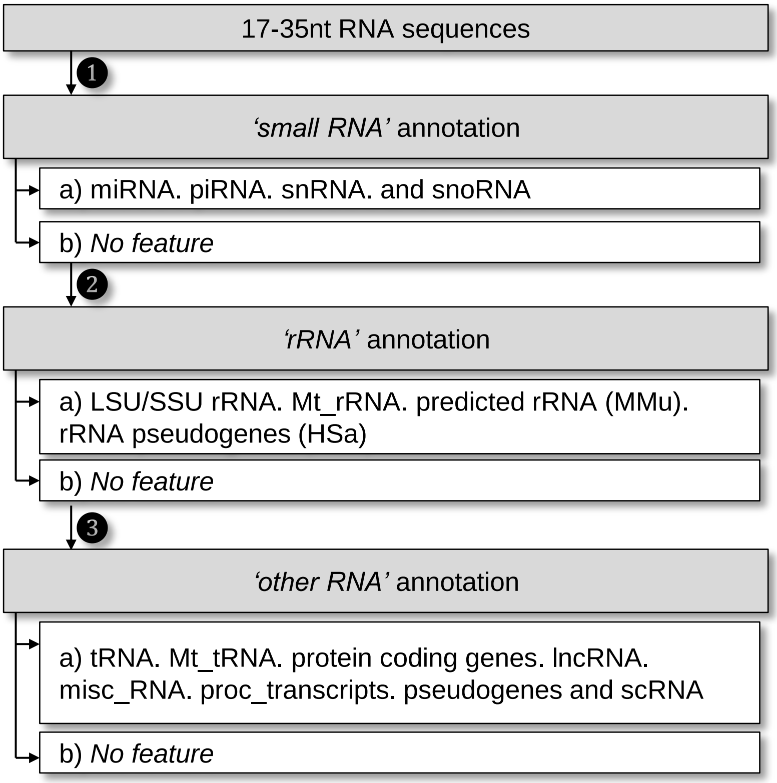

Supplementary Figure S2. Annotation of small RNA sequencing data. All mapped reads in the size range of 17–35 nt reads were annotated against the respective human or mouse 'small RNA' genomic features (gene biotypes according to Vega, version 68). The reads with no found 'small RNA' feature were filtered out and subjected to 'rRNA' annotation. Finally, all reads that did not match 'small RNA' or 'rRNA' were annotated against the custom-compiled list of 'other RNA' annotations. LSU/SSU rRNA – large/small subunit ribosomal RNA; Mt_rRNA – mitochondrial ribosomal RNA; misc_RNA – miscalleous RNA (includes Y RNA); proc_transcripts – processed transcripts. scRNA - small cytoplasmic RNA.

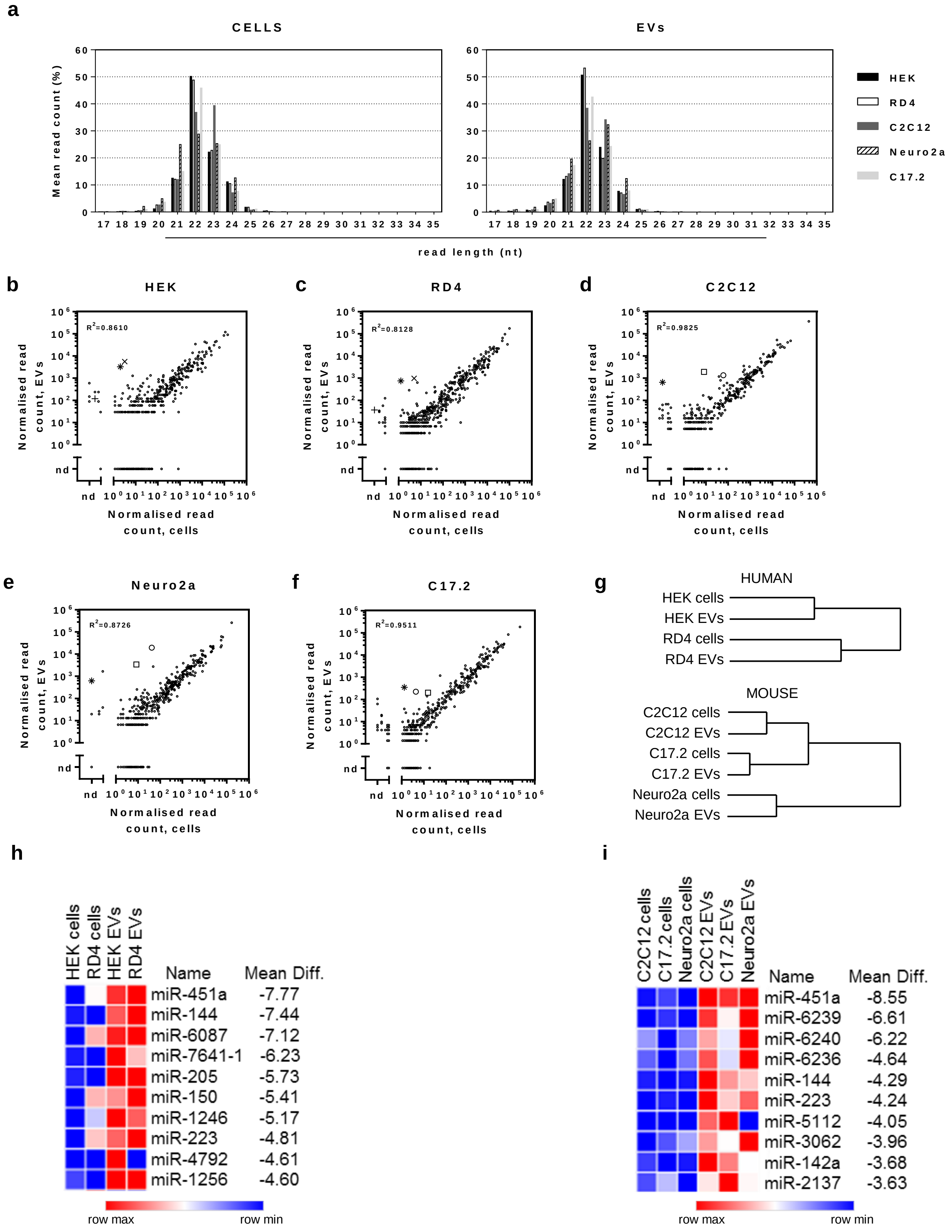

Supplementary Figure S3. Length distribution, correlation and clustering of vesicular and cellular miRNAs. (a) Length distribution of miRNA reads indicates to the prominence of mature miRNA sequences. Normalised miRNA read count in HEK **(b),** RD4 **(c)**, C2C12 **(d),** Neuro2a **(e)** and C17.2 **(f)** miRNA expression levels in EVs correlate with levels in parental cells. A small number of individual miRNAs deviated from the correlation (few selected samples are labelled in panels A-E). **(g)** Hierarchical clustering of log2 transformed normalized miRNA counts showed that EVs cluster together with their parental cells rather than with EVs derived from other cell types. **(h-i)** Heatmaps of top 10 EV-enriched miRNAs within human **(h)** and mouse **(i)** samples considering the mean difference in normalised expression values between cells and EVs (on a relative scale per row/miRNA). MiRNAs with read count ≥ 3 were considered in the correlation and clustering analysis. * miR 451a; + miR-144; x miR-6087; o miR-6240 ; □ miR-6239.

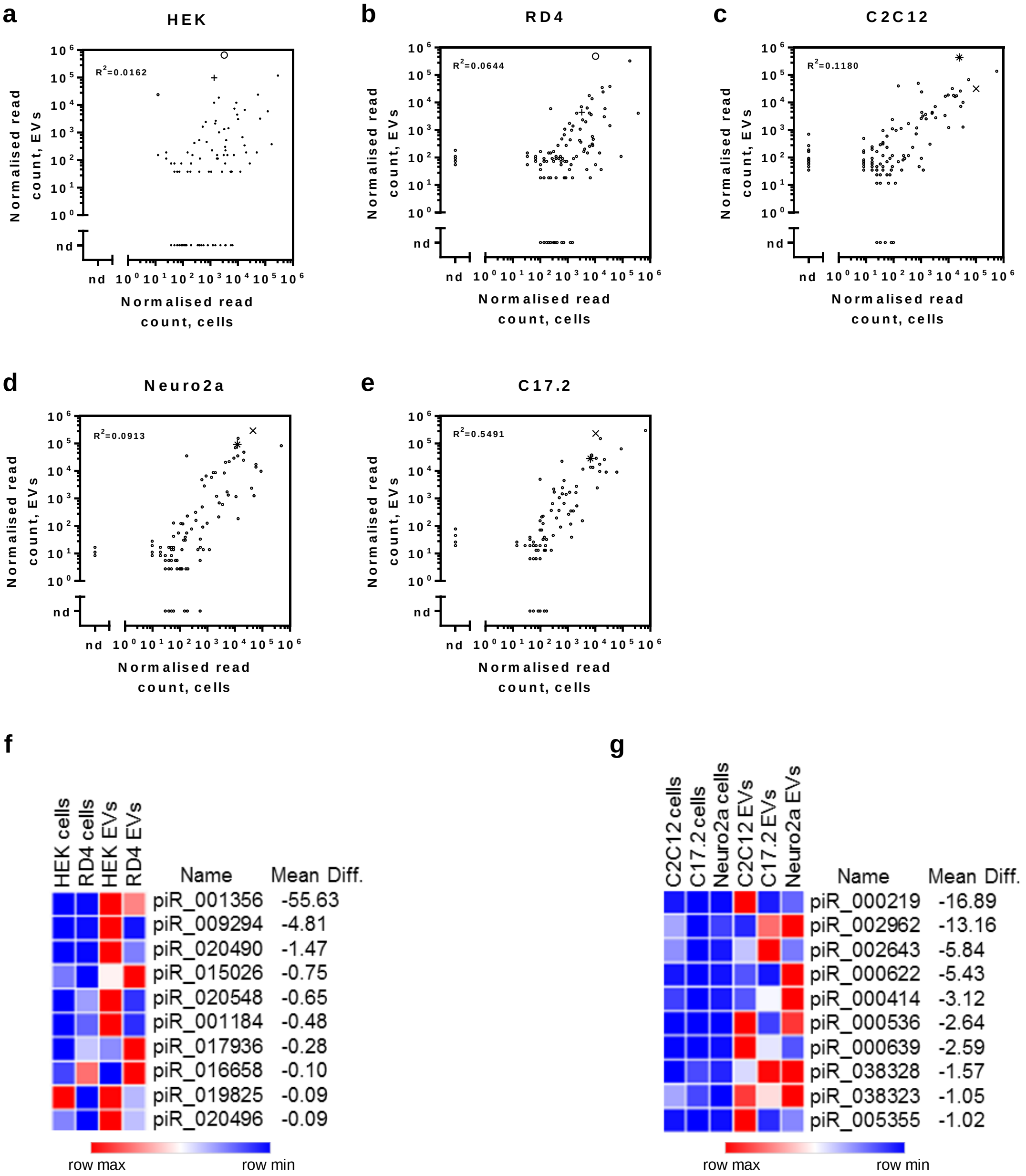

Supplementary Figure S4. Correlation of vesicular and cellular piRNA expression levels. Normalised piRNA read counts in HEK **(a)**. RD4 **(b)**. C2C12 **(c)**. Neuro2a **(d)** and C17.2 **(e)** EVs were very poorly correlated with their cellular expression levels. Heatmaps depicting the top 10 EV-enriched piRNAs within human **(f)** and mouse **(g)** samples. Mean difference in normalised expression values between cells and EVs (on a relative scale per row/piRNA). piRNAs with read count ≥ 3 were considered in the analysis. o piR_001356 (DQ571873); + piR_009294 (DQ582566); x piR_002962 (DQ548183); * piR_000219 (DQ540058).

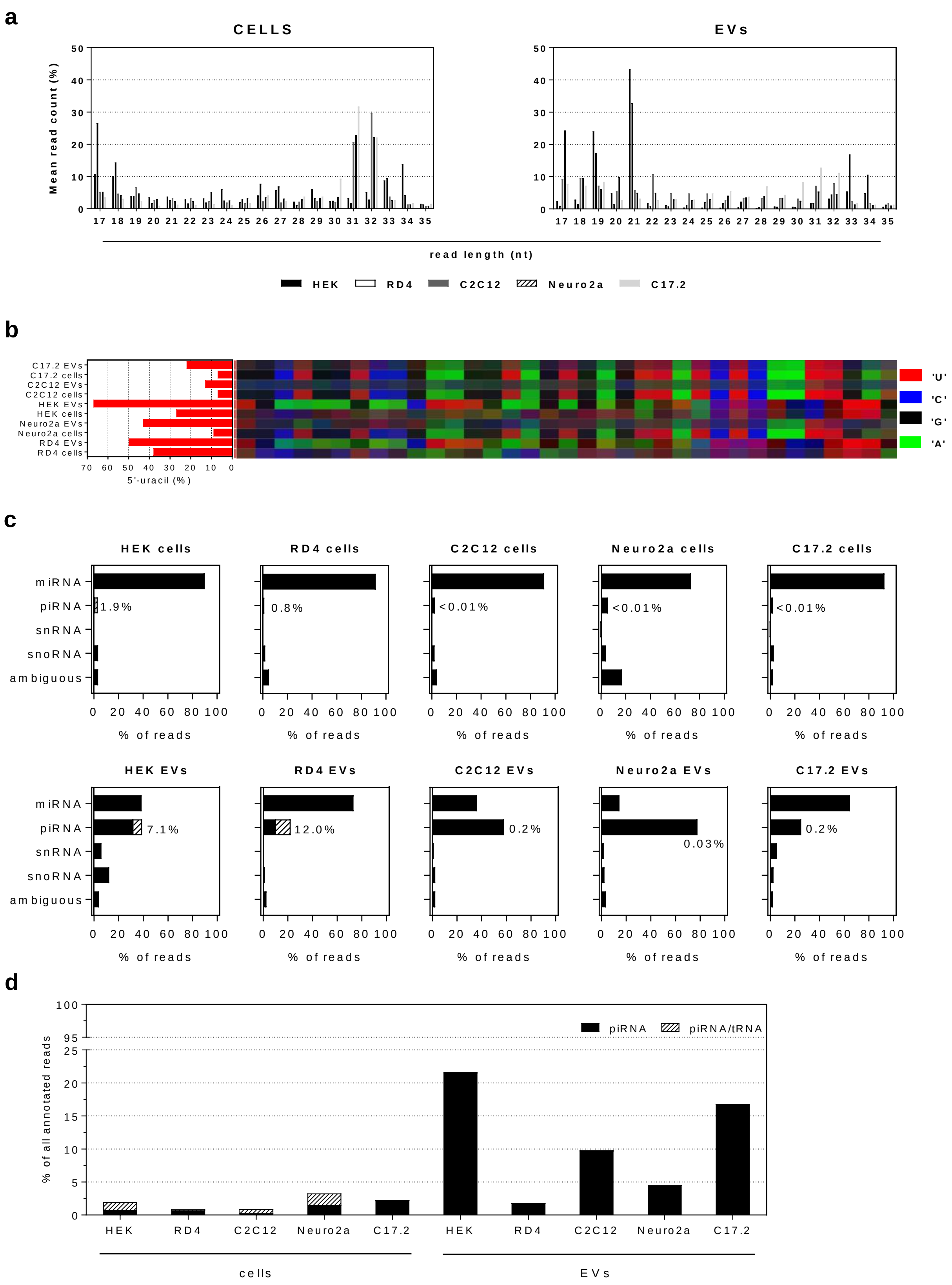

Supplementary Figure S5. Characterization of reads mapping to piRNA loci. Limited number of reads mapping to piRNA loci were detected in the 27-35 nt size range **(a)** and displayed the preference to 5'-uracil **(b)**, being more common for vesicular than cellular piRNA annotations. Though some piRNA annotations can potentially represent tRNA fragments (striped area of the piRNA bar with % denoting its contribution to 'small RNA' category) **(c)**, this was seen to have a low to negligible influence on cellular and EV samples, respectively (up to 1.8% and up to 0.09% ambiguous piRNA/tRNA annotations; striped area based on the total number of annotated reads) **(d)**. The heatmap illustrates the relative base composition of piRNA reads in 5' → 3' orientation. '% of reads' in (c) denotes the contribution of individual biotypes within the 'small RNA' category.

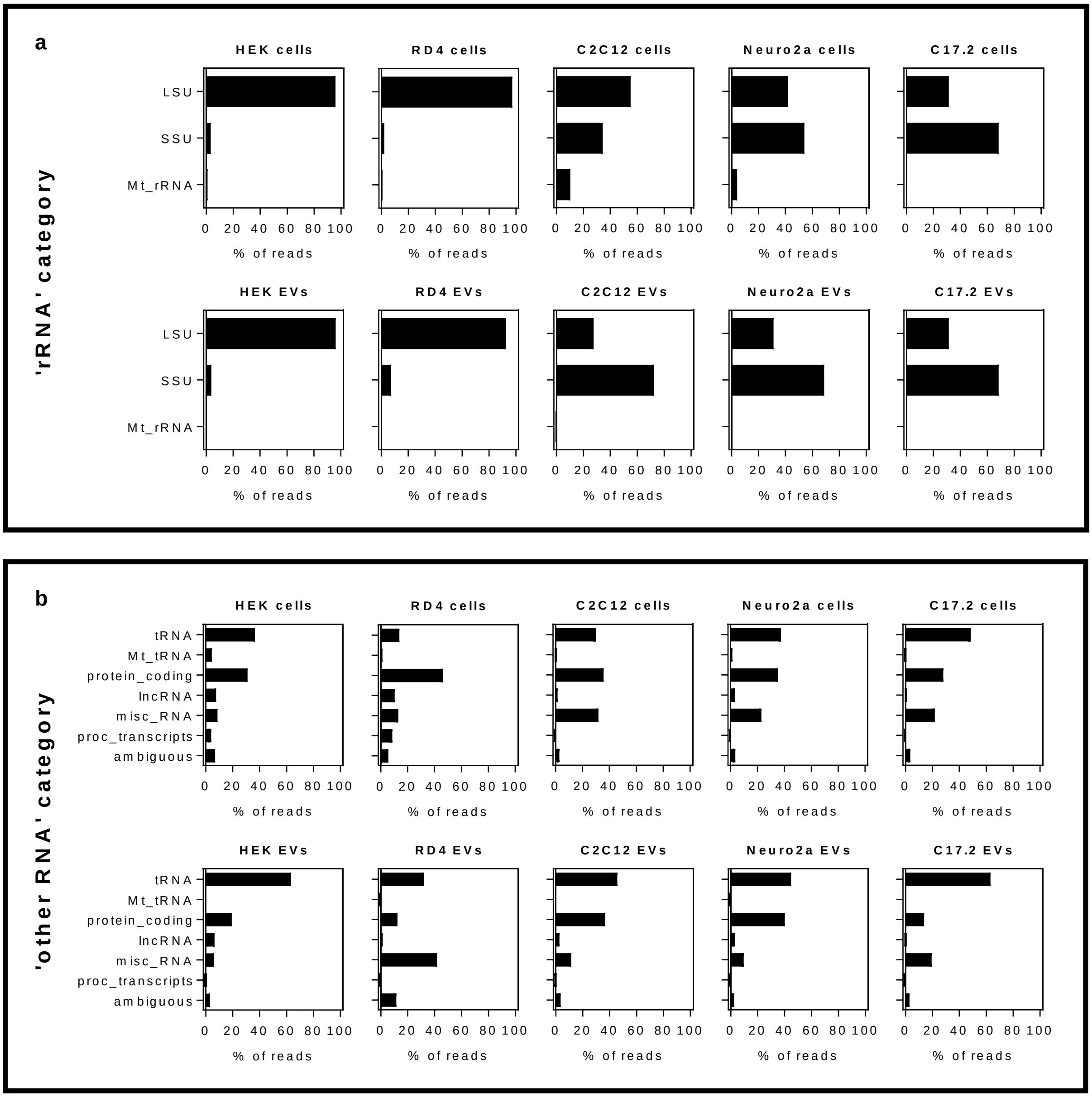

Supplementary Figure S6. Contribution of individual gene biotypes to the ‘rRNA’ and ‘other RNA’ categories. (a) Human-derived EVs and parental cells contained mostly fragments from the large ribosomal subunit, whereas mouse cell-lines also held many fragments from the small subunit, the latter being more emphasized for EV- than cell samples. **(b)** The ‘other RNA’ category was found enriched in fragments from tRNAs, protein coding genes and miscallenous RNA. This subclass also included gene biotypes of pseudogenes and small cytoplasmic RNA which in total constituted <2% of the subclass reads in cells and EVs and are not depicted on the graph. LncRNA – long non-coding RNA, misc_RNA – miscellaneous RNA (including e.g. Y RNA and vault RNA), proc_transcripts – processed transcripts, ambiguous – reads that were assigned to more than one genomic feature within the particular category. % of reads is denoted as the fraction of individual biotypes within the indicated category. The ‘rRNA’ category does not depict mouse predicted rRNA genes, human 5S and 5.8S pseudogenes and mouse/human ambiguous rRNA genes which covered <0.3%, <0.004% and < 0.07% of reads in the ‘rRNA’ category, respectively.

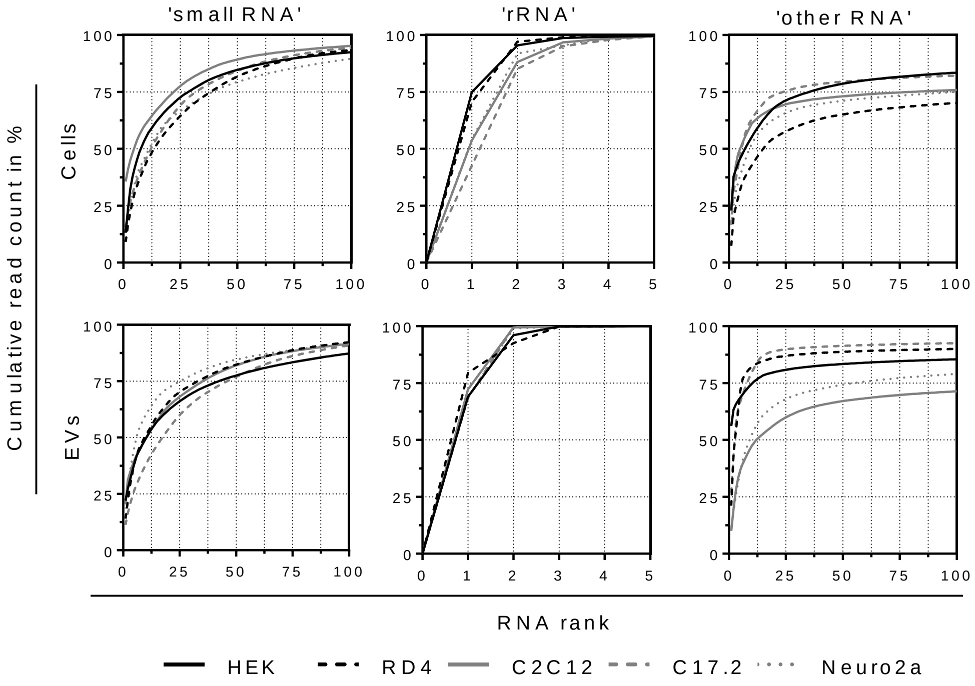

Supplementary Figure S7. Cumulative histograms describing the contribution of individual genes in the 'small RNA'. 'rRNA' and 'other RNA' categories throughout tested samples. For both 'small RNA' as well as 'other RNA' categories, only a small number of individual genes contributed to the majority (75%) of total RNA reads (and thus to total bulk mass of RNA). This was more pronounced for annotations in the 'other RNA' than 'small RNA' category and was most prominent for vesicular 'other RNA' where the majority of the effect could be attributed to the presence of tRNA- and Y RNA fragments.

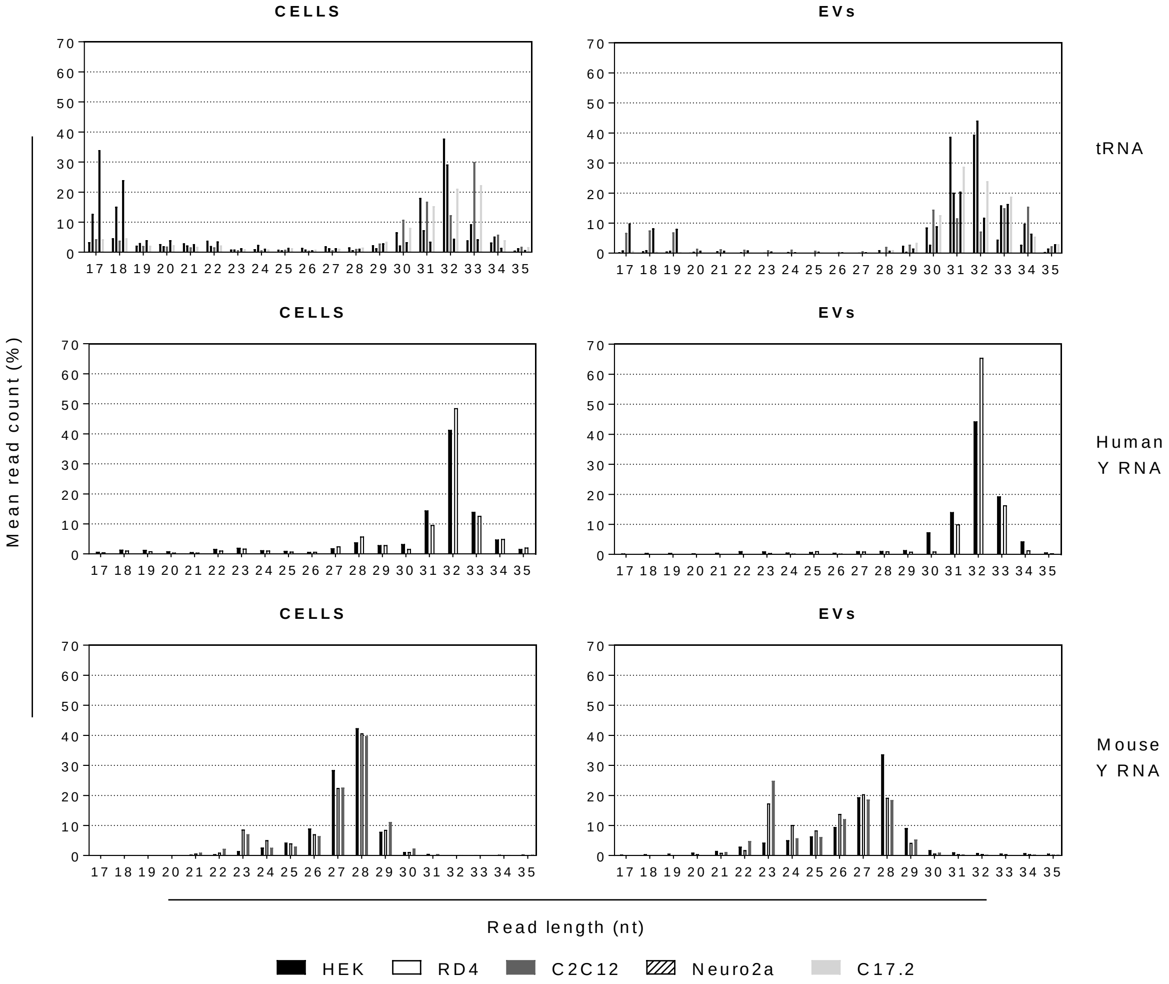

Supplementary Figure S8. Length distribution of tRNA and Y RNA reads. The majority of reads annotated as tRNA fell in the size range of 30-34 nt, showing no differences between cellular and vesicular samples. Though the bulk of human Y RNA reads displayed a similar size profile as tRNA sequences, the majority of mouse Y RNA reads were shorter (26-29 nt).

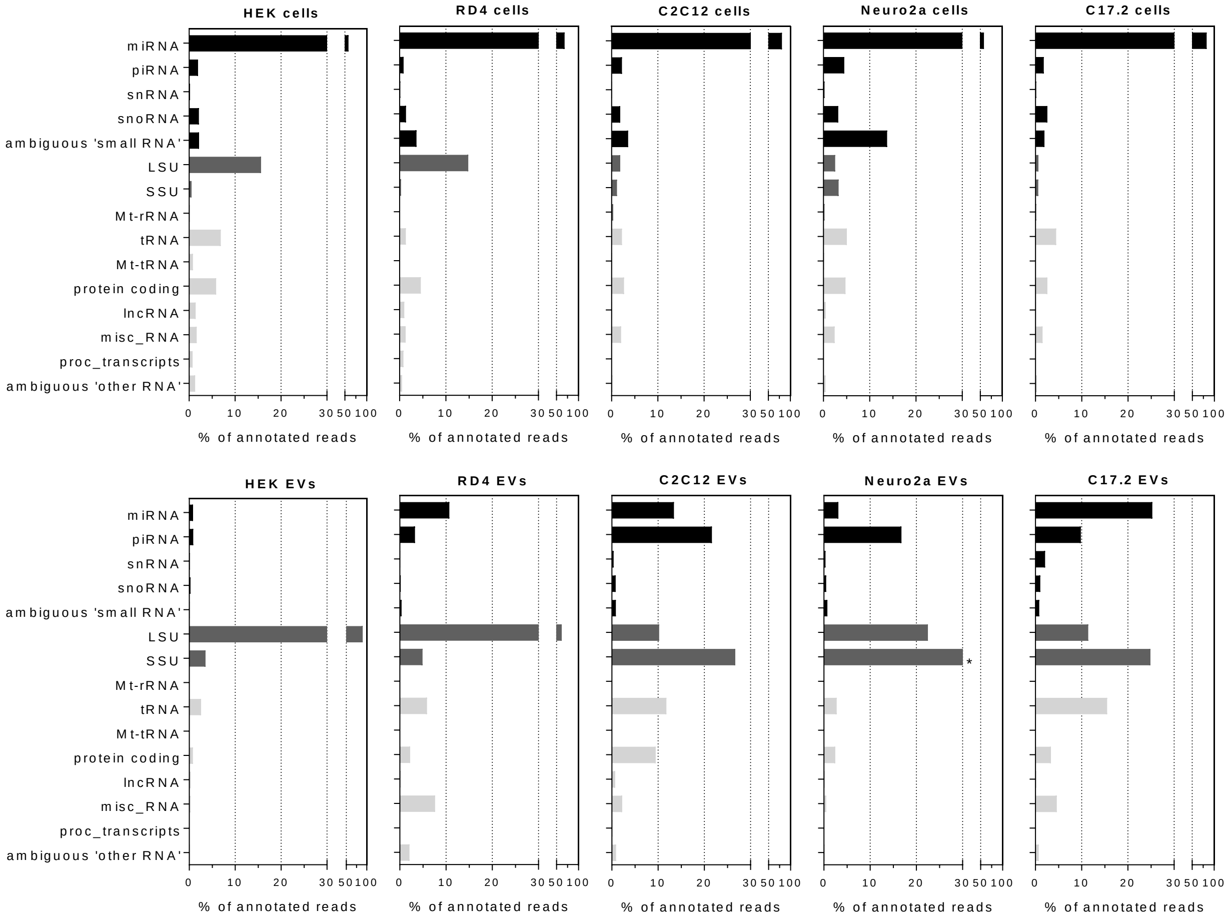

Supplementary Figure S9. Overall contribution of 'small RNA' (black), 'rRNA' (dark grey) and 'other RNA' (light grey) gene biotypes to the total pool of annotated reads. The most prominent biotype in cells was miRNAs, followed by large subunit of rRNA, tRNA and protein coding RNA. In contrast, EV samples were extremely rich in rRNA fragments with modest to low content of miRNA, yet reflecting the cellular tendency of containing tRNA and protein coding RNA fragments. LSU rRNA– large subunit ribosomal RNA, SSU rRNA – small subunit ribosomal RNA, lncRNA – long non-coding RNA, misc_RNA – miscellaneous RNA), proc_transcripts – processed transcripts, ambiguous – reads that were assigned to more than one genomic feature within the particular category. % of reads is denoted as the fraction of individual biotypes within the total pool of annotated reads. * - denotes 49.6% for SSU for Neuro2a EVs which is not visible since the x-axis has been cut. Predicted rRNA genes (MMu), rRNA pseudogenes (HSa) and small cytoplasmic RNA constituting <0.04%, <0.5% and <0.0003% of the annotated reads, respectively are not depicted on the graphs.

C2C12 EVs

GO molecular function complete	Fold enrichment	P value
virus receptor activity	33.5	5.4E-03
threonine-type endopeptidase activity	6.7	2.5E-04
structural constituent of cytoskeleton	6.1	3.9E-07
unfolded protein binding	5.1	6.5E-06
GTPase activity	4.0	9.1E-11
structural constituent of ribosome	3.7	3.0E-06
GTP binding	3.2	1.0E-09
Unclassified	0.6	0.0E+00
transferase activity	0.3	6.8E-04
**GO biological process complete**	**Fold enrichment**	**P value**
tumor necrosis factor-mediated signaling pathway	> 100	2.4E-19
NIK/NF-kappaB signaling	> 100	2.4E-19
regulation of cellular amino acid metabolic process	> 100	2.7E-17
Wnt signaling pathway, planar cell polarity pathway	71.2	7.5E-23
SRP-dependent cotranslational protein targeting to membrane	60.7	1.2E-38
anaphase-promoting complex-dependent catabolic process	54.5	1.8E-15
cell cycle phase	54.5	1.8E-15
positive regulation of protein ubiquitination involved in ubiquitin-dependent protein catabolic process	54.5	1.8E-15
Fc receptor mediated stimulatory signaling pathway	50.3	8.1E-06
regulation of cellular response to heat	50.3	8.1E-06
canonical glycolysis	41.9	4.2E-04
antigen processing and presentation of exogenous peptide antigen	39.1	7.4E-15
negative regulation of ubiquitin-protein transferase activity	36.3	3.1E-13
innate immune response-activating signal transduction	36.3	3.1E-13
nuclear-transcribed mRNA catabolic process, nonsense-mediated decay	34.7	1.0E-31
viral gene expression	34.7	1.0E-31
retina homeostasis	33.5	1.8E-02
antigen processing and presentation of peptide antigen via MHC class I	25.1	3.4E-10
response to vitamin	25.1	4.7E-04
neural nucleus development	25.1	4.7E-04
extracellular matrix disassembly	22.3	1.0E-05
positive regulation of ubiquitin-protein transferase activity	21.8	1.9E-10
midbrain development	19.6	2.4E-04
negative regulation of canonical Wnt signaling pathway	19.6	8.9E-11
T cell receptor signaling pathway	18.2	1.8E-09
G2/M transition of mitotic cell cycle	12.6	2.5E-02
regulation of mRNA stability	10.2	5.0E-09
positive regulation of canonical Wnt signaling pathway	9.8	7.8E-07
positive regulation of protein localization to Cajal body	8.4	1.6E-02
translational initiation	7.4	2.5E-13
leukocyte migration	7.3	2.9E-05
MAPK cascade	6.4	4.5E-04
rRNA processing	6.1	3.2E-11
protein polyubiquitination	5.2	4.6E-03
single fertilization	5.1	3.2E-02
blood coagulation	4.0	3.1E-02
protein folding	4.0	1.2E-05
regulation of mitotic cell cycle phase transition	3.8	3.0E-02
RNA biosynthetic process	3.0	2.2E-05
small GTPase mediated signal transduction	2.7	3.9E-03
organelle organization	1.6	2.3E-02
Unclassified	0.2	0.0E+00
**GO cellular component complete**	**Fold enrichment**	**P value**
melanosome	27.93	6.2E-20
vesicle lumen	13.96	1.9E-02
endocytic vesicle membrane	12.57	5.7E-03
endoplasmic reticulum lumen	10.05	2.0E-02
chaperonin-containing T-complex	8.38	9.7E-04
zona pellucida receptor complex	7.45	8.5E-03
proteasome core complex	6.28	3.8E-04
cytosolic small ribosomal subunit	5.49	1.7E-06
blood microparticle	4.33	1.5E-03
endoplasmic reticulum membrane	3.63	4.3E-02
Golgi membrane	3.51	2.9E-03
extracellular matrix	3.07	1.1E-12
myelin sheath	2.52	4.0E-03
focal adhesion	2.37	3.3E-07
polymeric cytoskeletal fiber	2.24	3.6E-03
extracellular exosome	1.52	2.3E-11
Unclassified	0.44	0.0E+00

Supplementary Figure S10. Overrepresented Gene Ontology (GO) classes of the most abundant proteins in C2C12 EV proteome. The top 75th percentile of the most abundant C2C12 EV proteins was highly enriched in proteins related to various signalling pathways (e.g. TNF, NIK/NF-κB, Wnt, MAPK), cell cycle regulation as well as endoplasmatic reticulum related processes (e.g. amino acid metabolic- and protein ubiquitination processes, protein targeting to membrane). Results from Panther Statistical Overrepresentation Test by using a reference list of all protein annotations from C2C12 EVs.

HEK EVs

GO molecular function complete	Fold enrichment	P value
structural constituent of ribosome	10.3	6.0E-47
rRNA binding	7.8	7.1E-10
unfolded protein binding	3.9	1.6E-02
Unclassified	2.2	0.0E+00
poly(A) RNA binding	2.0	8.7E-11
catalytic activity	0.7	2.1E-03
ion binding	0.5	4.6E-02

GO biological process complete	Fold enrichment	P value
ribosomal small subunit assembly	10.6	3.9E-05
SRP-dependent cotranslational protein targeting to membrane	10.3	8.1E-45
viral transcription	9.9	9.4E-44
nuclear-transcribed mRNA catabolic process, nonsense-mediated decay	8.8	1.7E-40
translational initiation	7.0	1.3E-35
single fertilization	6.9	2.6E-02
ribosomal large subunit assembly	6.5	4.3E-02
cytoplasmic translation	5.6	1.1E-03
rRNA processing	5.0	2.7E-26
Unclassified	1.8	0.0E+00
regulation of biological process	0.8	3.0E-02

GO cellular component complete	Fold enrichment	P value
cytosolic small ribosomal subunit	9.9	8.5E-18
cytosolic large ribosomal subunit	9.3	3.9E-24
nucleosome	7.7	9.1E-03
blood microparticle	3.3	2.8E-02
extracellular matrix	3.1	1.0E-08
focal adhesion	2.9	9.9E-09
Unclassified	2.6	0.0E+00
extracellular exosome	1.6	8.8E-09
endomembrane system	0.6	1.2E-02
organelle membrane	0.5	2.5E-02

Supplementary Figure S11. Overrepresented Gene Ontology (GO) classes of the most abundant proteins in HEK EVs. Proteins belonging to the top 75th percentile of the HEK EV proteome were highly enriched in ribosome-related GO terms, exceeding 10 times their expected background levels. Additionally, a high enrichment of proteins related to membrane targeting and viral transcription was detected, the latter reflecting the SV40 large T antigen transduced nature of the parental cells. Results from Panther Statistical Overrepresentation Test by using a reference list of all protein annotations from HEK EVs.

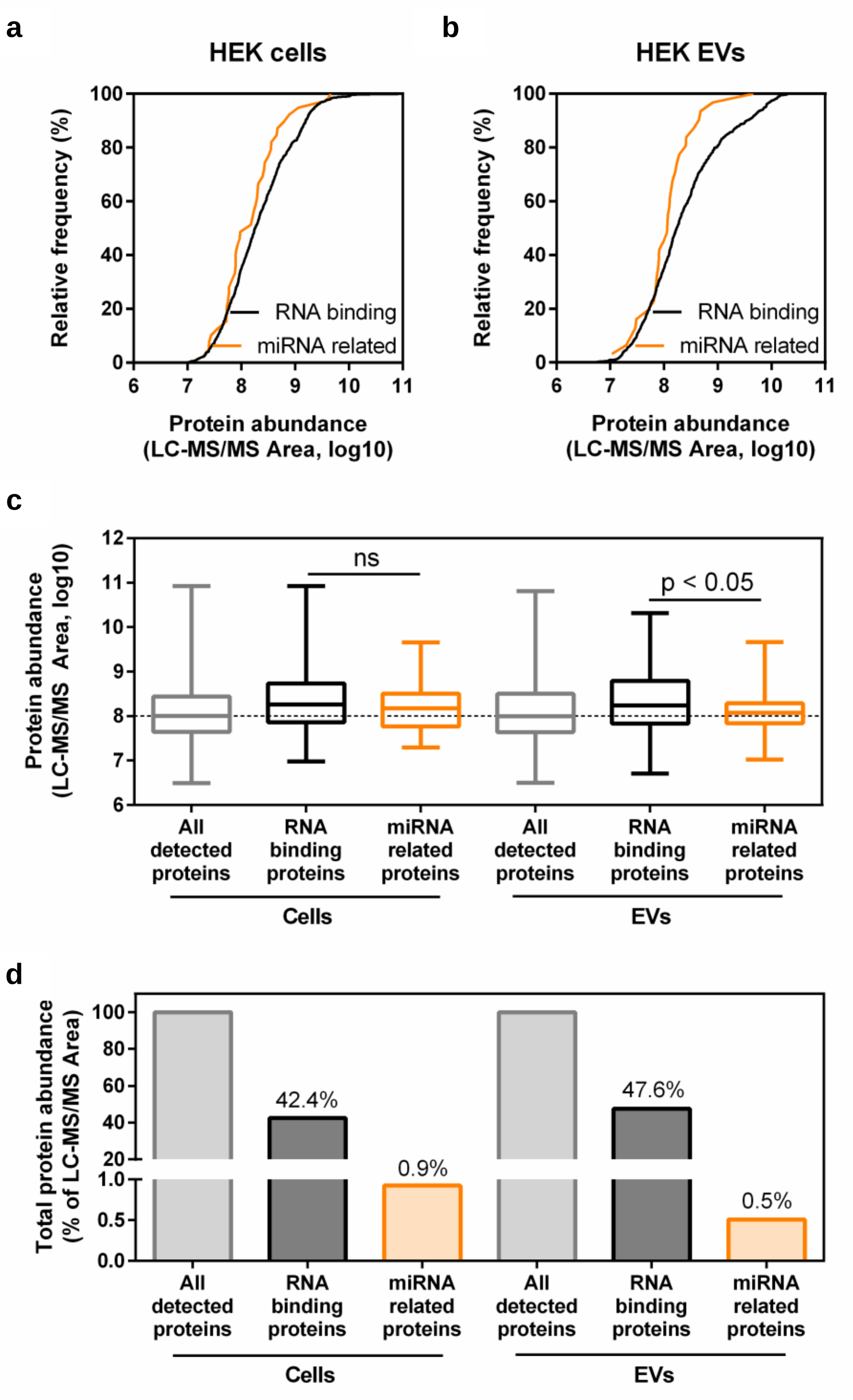

Supplementary Figure S12. The comparison of RNA binding proteins and miRNA related proteins within the proteome of EVs as well as their source HEK cells. Cumulative relative frequency distribution was calculated for **(a)** the cell proteome as well as for **(b)** the EV proteome. **(c)** Statistical analysis of these cumulative frequency distributions (using the Kolmogorov-Smirnov nonparametric test) revealed that in cells the distribution of RNA binding proteins and miRNA related proteins is similar, however in EV samples, miRNA related proteins are more likely to be found among the proteins with lower expression, as compared to the overall distribution of all RNA binding proteins. **(d)** miRNA related proteins comprise of approx. 0.9% of total protein mass in cell samples, whereas in EV samples there is a trend of the total amount miRNA related proteins being nearly two times lower than in corresponding cell samples.

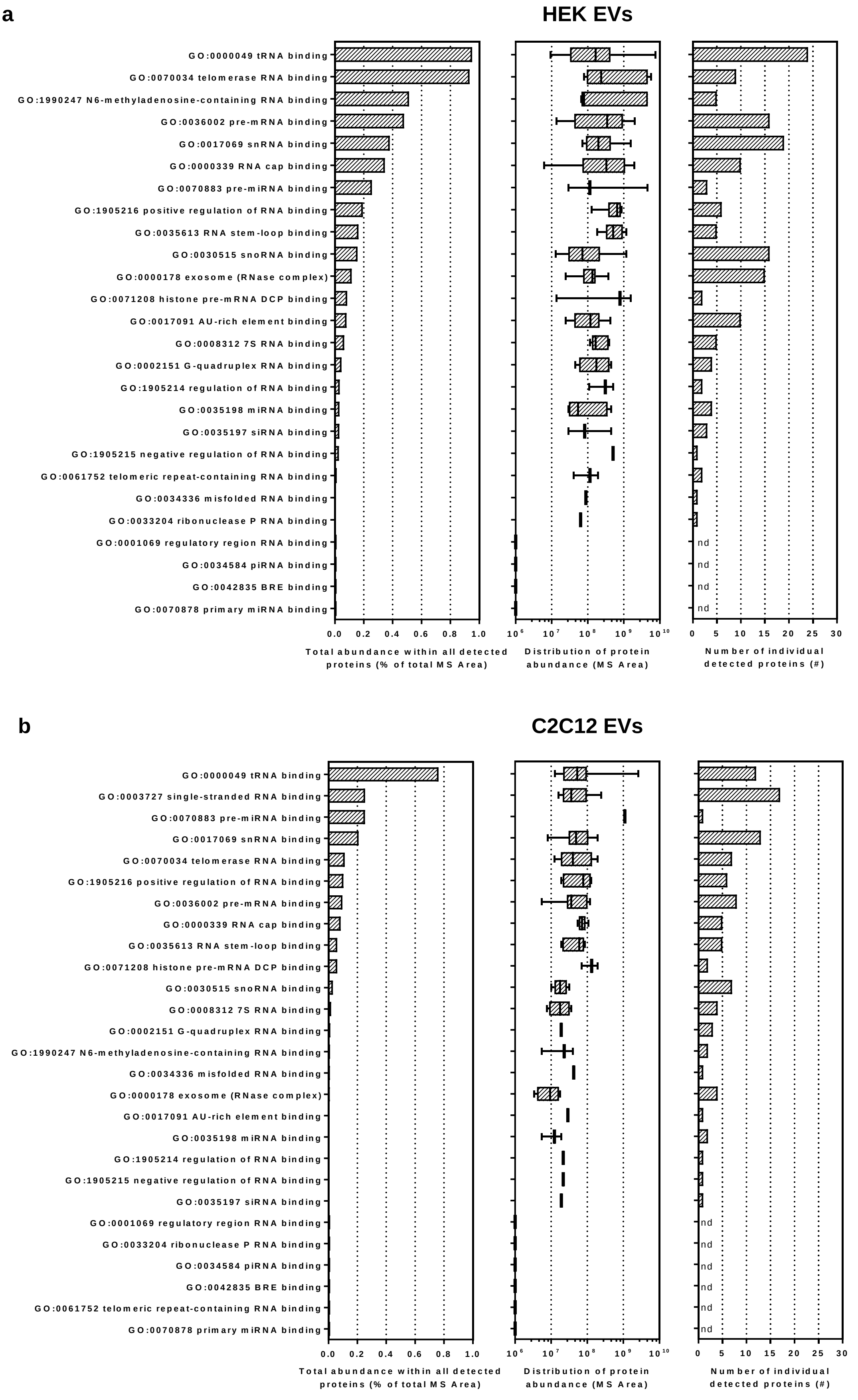

Supplementary Figure S13. Low-abundant GO term based RNA binding protein sets of HEK- (a) and C2C12 EVs (b). The graphs depict ‘RNA binding’ GO:0003723 child terms contributing <1% of total protein mass in the EVs samples. In some cases (e.g. GO:1990247 for HEK EVs), already a low number of highly abundant proteins were able to contribute to the EV proteome to the same extent as numerous proteins categorized under other GO terms (GO:0036002 for HEK EVs). Please note the scale in the total protein abundance (% of total MS Area) goes until 1%. GO classes with >1% contribution to the respective proteomes can be found in Figure 6. nd – not detected.

Supplementary Table S1. Overview of the included gene biotypes in the 'small RNA'. 'rRNA' and 'other RNA' categories. All mapped reads in the size range of 17–35 nt reads were sequentially annotated against the respective human or mouse genomic features classified under 'small RNA', 'rRNA' or 'other RNA' categories as specified below. NA – not applicable.

<table>
<tr><th>Category</th><th>Transcript biotype</th><th>Transcript subclassification</th><th>Annotation source</th></tr>
<tr><td rowspan="4">'small RNA'</td><td>miRNA</td><td>miRNA_primary_transcript</td><td>miRBase release 21</td></tr>
<tr><td>piRNA</td><td>NA</td><td>piRNAbank 38 (UCSC liftOver to genome build 38 coordinate system)</td></tr>
<tr><td>snRNA</td><td>NA</td><td>Ensembl 38.85</td></tr>
<tr><td>snoRNA</td><td>NA</td><td>Ensembl 38.85</td></tr>
<tr><td rowspan="2">'rRNA'</td><td>rRNA</td><td>LSU_rRNA. SSU_rRNA. predicted rRNA genes (MMu). 5S/5.8S rRNA pseudogenes (HSa)</td><td>UCSC Table Browser Hg38/Mm10 RepeatMasker rRNA entries + Ensembl 38.85 rRNA annotations</td></tr>
<tr><td>Mt_rRNA</td><td>NA</td><td>Ensembl 38.85</td></tr>
<tr><td rowspan="22">'other RNA'</td><td>tRNA</td><td>NA</td><td>UCSC Table Browser</td></tr>
<tr><td>Mt_tRNA</td><td>NA</td><td rowspan="21">Ensembl 38.85</td></tr>
<tr><td>protein_coding</td><td>NA</td></tr>
<tr><td rowspan="7">lncRNA</td><td>3prime_overlapping_ncRNA</td></tr>
<tr><td>antisense</td></tr>
<tr><td>bidirectional_promoter_lncRNA</td></tr>
<tr><td>lincRNA</td></tr>
<tr><td>macro_lncRNA</td></tr>
<tr><td>sense_intronic</td></tr>
<tr><td>sense_overlapping</td></tr>
<tr><td>misc_RNA</td><td>NA</td></tr>
<tr><td>processed_transcript</td><td>NA</td></tr>
<tr><td rowspan="9">pseudogenes</td><td>polymorphic_pseudogene</td></tr>
<tr><td>processed_pseudogene</td></tr>
<tr><td>pseudogene</td></tr>
<tr><td>transcribed_processed_pseudogene</td></tr>
<tr><td>transcribed_unitary_pseudogene</td></tr>
<tr><td>transcribed_unprocessed_pseudogene</td></tr>
<tr><td>unitary_pseudogene</td></tr>
<tr><td>unprocessed_pseudogene</td></tr>
<tr><td>scRNA</td><td>NA</td></tr>
</table>

Supplementary Table S2. Abundance rank of potential fetal bovine serum (FBS) confounding miRNAs. Four FBS-confounding miRNAs reported by Wei *et al.* (1) were checked for abundance in cell- and EV samples. MiRNAs which had a higher abundance in EVs as opposed to cells and rank under top 100 most abundant miRNAs within the sample pair (cells + EVs) are marked with grey-shaded entries. Abundance ranking is based on the list of unique miRNA identifications, with the highest rank denoting the highest abundance level of a particular miRNA. Please note that the miRNAs reported by Wei *et al*. represent the mature miRNAs, whereas the ranking in the present table is based on miRNA annotations to predicted stem-loop structure (as performed throughout the study, see Supplementary Table S1 for more information).

miRNA	HEK		RD4		C2C12		Neuro2a		C17.2	
	Cells	EVs	Cells	EVs	Cells	EVs	Cells	EVs	Cells	EVs
miR-122	376	84	792	226	NA	NA	NA	NA	NA	NA
miR-1246	208	168	88	99	16	13	114	118	20	19
miR-148a	3	3	146	95	52	56	285	185	34	54
miR-451a	567	49	648	119	565	113	708	128	405	137

Supplementary Table S3. Top 20 detected miRNAs in all analysed EVs and their parental cells. The top 20 EV-associated miRNAs overlapped largely with the top 20 miRNAs of the respective source cell, with some exceptions (grey-shaded entries). Throughout EV samples miR-21, miR-92a-1, miR-378a, miR-30d, and miR-99b were the most commonly identified miRNAs, appearing in four fifths of all such samples (underlined entries).

	HEK		RD4		C2C12		C17.2		Neuro2a	
**Rank**	**Cells**	**EVs**	**Cells**	**EVs**	**Cells**	**EVs**	**Cells**	**EVs**	**Cells**	**EVs**
1	miR-10a	miR-92a-1	miR-100	miR-100	miR-21a	miR-21a	miR-21a	miR-21a	miR-21a	miR-21a
2	miR-92a-1	miR-10a	miR-21	miR-206	miR-22	miR-140	let-7i	miR-27b	miR-27b	miR-143
3	miR-148a	miR-148a	miR-206	miR-92a-1	miR-143	miR-22	miR-27b	let-7i	miR-218-2	miR-27b
4	miR-218-1	miR-92a-2	miR-92a-1	miR-99a	let-7i	miR-143	let-7f-2	let-7f-2	miR-143	miR-7a-2
5	miR-92a-2	miR-378a	miR-99a	miR-21	let-7f-2	miR-378a	miR-22	miR-140	miR-7a-2	miR-218-2
6	miR-10b	miR-10b	miR-532	miR-92a-2	miR-206	miR-99b	miR-99a	let-7g	miR-218-1	miR-92a-1
7	miR-20a	miR-221	miR-10a	miR-320a	miR-27b	let-7i	miR-143	miR-22	miR-7a-1	miR-30d
8	miR-218-2	miR-93	miR-183	miR-423	miR-378a	let-7c-2	let-7g	miR-92a-1	miR-30d	miR-99b
9	miR-21	miR-20a	miR-26a-1	miR-30d	miR-199a-2	let-7f-2	miR-99b	miR-99a	miR-470	miR-218-1
10	miR-30d	miR-218-1	miR-30d	miR-10a	miR-99b	let-7c-1	let-7c-2	miR-99b	miR-22	miR-22
11	miR-423	miR-423	miR-1307	miR-532	let-7c-2	let-7g	miR-26a-2	miR-26a-1	let-7c-2	miR-6240
12	miR-221	miR-30d	miR-92a-2	miR-99b	let-7c-1	miR-27b	miR-26a-1	miR-26a-2	let-7c-1	miR-7a-1
13	miR-222	miR-21	miR-99b	miR-378a	miR-140	let-7b	let-7c-1	let-7c-2	miR-24-2	miR-470
14	miR-26a-1	miR-26a-1	miR-27b	miR-183	let-7g	miR-199a-2	miR-24-2	let-7c-1	miR-99b	let-7c-2
15	miR-128-1	miR-25	miR-423	miR-1307	let-7f-1	miR-206	miR-92a-1	miR-24-2	miR-24-1	let-7c-1
16	miR-378a	miR-191	let-7i	miR-483	let-7b	miR-100	let-7f-1	miR-23a	miR-92a-1	miR-23b
17	miR-17	miR-186	let-7a-3	miR-26a-1	miR-30d	miR-99a	miR-24-1	miR-143	miR-128-1	miR-378a
18	miR-99a	let-7g	miR-378a	miR-221	miR-24-2	miR-24-2	miR-183	miR-24-1	miR-126a	miR-24-2
19	miR-7-3	miR-222	miR-320a	miR-25	miR-99a	miR-24-1	miR-30a	let-7b	miR-26a-1	miR-24-1
20	miR-7-2	miR-218-2	let-7g	miR-27b	miR-24-1	miR-30d	let-7b	miR-30a	miR-26a-2	miR-878

Supplementary Table S4. Top 20 annotations of RNA genes in all analysed EVs and their parental cells. Unlike parental cells. vesicle samples were rich in rRNA fragments as well as sequences derived from piRNA-, tRNA and Y RNA encoding loci. The relative contribution (percentage) per annotated gene was calculated over the total pool of annotated reads within each sample. After depicting the top 20 table of genes, a three-step colour scheme was appended whereby the maximum and minimum values in the table were taken as endpoints of the colour scheme, allowing the gene expression levels to be compared between different samples in the table.

Rank	HEK		RD4		C2C12		C17.2		Neuro2a	
	**Cells**	**EVs**	**Cells**	**EVs**	**Cells**	**EVs**	**Cells**	**EVs**	**Cells**	**EVs**
1	LSU-rRNA	LSU-rRNA	LSU-rRNA	LSU-rRNA	miR-21a	SSU-rRNA	let-7i	SSU-rRNA	miR-21a	SSU-rRNA
2	miR-10a	SSU-rRNA	miR-100	SSU-rRNA	miR-22	LSU-rRNA	miR-27b	LSU-rRNA	miR-27b	LSU-rRNA
3	miR-92a-1	tRNA-Gly-GCC	miR-21	tRNA-Gly-GCC	miR-143	miR-21a	let-7f-2	tRNA-Glu-CTC	SSU-rRNA	piR_002962
4	miR-148a	piR_001356	miR-206	Y_RNA	let-7i	tRNA-His-GTG	miR-22	tRNA-Gly-GCC	miR-218-2	piR_000622
5	tRNA-Gly-GCC	RMRP	miR-92a-1	miR-100	let-7f-2	tRNA-Gly-GCC	miR-99a	miR-21a	miR-143	miR-21a
6	miR-218-1	miR-92a-1	miR-99a	RNY4	miR-206	piR_000219	miR-143	Rny1	miR-7a-2	tRNA-Glu-CTC
7	miR-92a-2	tRNA-His-GTG	miR-532	RNY4P10	miR-27b	tRNA-Lys-CTT	let-7g	miR-27b	miR-218-1	piR_000219
8	miR-10b	piR_004987	miR-10a	RNY1	LSU-rRNA	tRNA-Glu-CTC	miR-99b	tRNA-Val-CAC	LSU-rRNA	piR_000935
9	miR-20a	miR-10a	miR-183	miR-206	Rny1	Rny1	let-7c-2	piR_000935	Rny1	piR_000414
10	miR-218-2	miR-148a	miR-26a-1	piR_001356	miR-378a	piR_000935	miR-26a-2	let-7i	miR-7a-1	tRNA-His-GTG
11	miR-21	tRNA-Glu-TTC	miR-30d	tRNA-His-GTG	miR-199a-2	tRNA-Lys-TTT	miR-26a-1	piR_002962	miR-30d	tRNA-His-GTG
12	KLHL4	piR_009294	miR-1307	miR-92a-1	miR-99b	miR-140	let-7c-1	let-7f-2	miR-470	Klhl32
13	miR-30d	RNA5-8SP6	miR-92a-2	piR_004987	SSU-rRNA	miR-22	miR-24-2	miR-140	miR-22	Rny1
14	miR-423	Y_RNA	miR-99b	miR-99a	let-7c-2	Rn7s1	tRNA-Glu-CTC	tRNA-His-GTG	let-7c-2	piR_002643
15	miR-221	LASP1	miR-27b	tRNA-Val-CAC	let-7c-1	Hydin	miR-92a-1	let-7g	let-7c-1	tRNA-Gly-GCC
16	miR-222	RNY1	miR-423	miR-21	miR-140	Klhl32	let-7f-1	tRNA-Lys-CTT	miR-24-2	tRNA-Val-CAC
17	miR-26a-1	RNY4	let-7i	miR-92a-2	let-7g	tRNA-LeuAAG	miR-24-1	tRNA-Val-AAC	piR_000935	piR_000578
18	miR-128-1	RNY4P10	let-7a-3	miR-320a	let-7f-1	miR-143	miR-183	piR_002643	tRNA-Cys-GCA	piR_000536
19	miR-378a	AGAP1	miR-378a	tRNA-Glu-TTC	let-7b	miR-378a	miR-30a	miR-22	miR-99b	tRNA-Lys-CTT
20	miR-17	tRNA-Val-CAC	miR-320a	miR-423	miR-30d	miR-99b	let-7b	miR-92a-1	miR-24-1	piR_038328

Minimum of top 20 | 50th percentile | Maximum of top 20

Supplementary Table S5. Gene Ontology (GO) statistical enrichment analysis of C2C12 EV proteome using LOG10 transformed protein abundance values. Statistical enrichment was performed by employing Panther Statistical Enrichment Analysis with Bonferroni correction for multiple testing. Results were sorted hierarchically and only selected, statistically enriched GO subclass results are presented in this table. # number of proteins belonging to the respective GO class

**GO class/term (C2C12 EVs)**	**#**	**+/-**	**P value**
GO molecular function complete			
structural molecule activity (GO:0005198)	165	+	0.00E+00
guanyl ribonucleotide binding (GO:0032561)	122	+	6.57E-10
threonine-type peptidase activity (GO:0070003)	15	+	1.33E-03
GO biological process complete			
cotranslational protein targeting to membrane (GO:0006613)	63	+	0.00E+00
protein targeting to ER (GO:0045047)	64	+	0.00E+00
viral gene expression (GO:0019080)	66	+	0.00E+00
nuclear-transcribed mRNA catabolic process (GO:0000956)	80	+	2.83E-10
translation (GO:0006412)	135	+	1.15E-08
rRNA metabolic process (GO:0016072)	86	+	5.68E-05
wound healing (GO:0042060)	110	+	1.05E-04
ribosome biogenesis (GO:0042254)	92	+	1.52E-04
coagulation (GO:0050817)	68	+	4.17E-04
hemostasis (GO:0007599)	69	+	4.32E-04
non-canonical Wnt signaling pathway (GO:0035567)	58	+	4.38E-04
regulation of establishment of planar polarity (GO:0090175)	50	+	1.73E-03
midbrain development (GO:0030901)	15	+	7.38E-03
ncRNA processing (GO:0034470)	102	+	8.49E-03
regulation of cellular ketone metabolic process (GO:0010565)	44	+	2.55E-02
GO cellular component complete			
cytosolic ribosome (GO:0022626)	69	+	0.00E+00
small ribosomal subunit (GO:0015935)	29	+	1.10E-09
cell-substrate adherens junction (GO:0005924)	212	+	3.05E-09
extracellular vesicle (GO:1903561)	884	+	2.38E-08
extracellular region (GO:0005576)	1013	+	2.87E-07
extracellular space (GO:0005615)	216	+	2.41E-05
large ribosomal subunit (GO:0015934)	41	+	9.03E-05
pigment granule (GO:0048770)	60	+	7.58E-04
proteinaceous extracellular matrix (GO:0005578)	89	+	3.52E-03
extracellular matrix component (GO:0044420)	51	+	1.39E-02
intermediate filament (GO:0005882)	28	+	2.63E-02

Supplementary Table S6. Gene Ontology (GO) statistical enrichment analysis of HEK EV proteome using LOG10 transformed protein abundance values. Statistical enrichment was performed by employing Panther Statistical Enrichment Analysis with Bonferroni correction for multiple testing. Results were sorted hierarchically and only selected, statistically enriched GO subclass results are presented in this table. # number of proteins belonging to the respective GO class

GO class/term (HEK EVs)	#	+/-	P value
GO molecular function complete			
structural molecule activity (GO:0005198)	215	+	0.00E+00
poly(A) RNA binding (GO:0044822)	611	+	1.65E-12
RNA binding (GO:0003723)	737	+	7.15E-11
nucleoside-triphosphatase activity (GO:0017111)	289	+	3.71E-04
threonine-type peptidase activity (GO:0070003)	14	+	2.33E-03
GO biological process complete			
cotranslational protein targeting to membrane (GO:0006613)	76	+	0.00E+00
protein targeting to ER (GO:0045047)	77	+	0.00E+00
translation (GO:0006412)	176	+	0.00E+00
nuclear-transcribed mRNA catabolic process (GO:0000956)	127	+	0.00E+00
ribosome biogenesis (GO:0042254)	185	+	7.04E-13
rRNA metabolic process (GO:0016072)	165	+	7.04E-13
ncRNA processing (GO:0034470)	214	+	1.61E-07
regulation of RNA stability (GO:0043487)	83	+	1.01E-06
non-canonical Wnt signaling pathway (GO:0035567)	58	+	7.30E-06
regulation of establishment of planar polarity (GO:0090175)	53	+	3.19E-05
negative regulation of ubiquitin protein ligase activity (GO:1904667)	51	+	7.70E-05
regulation of cellular amine metabolic process (GO:0033238)	39	+	1.05E-04
regulation of ubiquitin-protein ligase activity involved in mitotic cell cycle (GO:0051439)	52	+	1.20E-04
antigen receptor-mediated signaling pathway (GO:0050851)	64	+	2.04E-04
regulation of cellular ketone metabolic process (GO:0010565)	45	+	2.19E-04
RNA splicing. via transesterification reactions with bulged adenosine as nucleophile (GO:0000377)	157	+	3.31E-04
positive regulation of Wnt signaling pathway (GO:0030177)	57	+	5.01E-04
cellular response to tumor necrosis factor (GO:0071356)	56	+	6.72E-04
positive regulation of ubiquitin protein ligase activity (GO:1904668)	53	+	7.81E-04
negative regulation of Wnt signaling pathway (GO:0030178)	55	+	2.78E-03
Fc receptor signaling pathway (GO:0038093)	85	+	3.80E-03
regulation of canonical Wnt signaling pathway (GO:0060828)	64	+	3.86E-03
antigen processing and presentation of exogenous peptide antigen via MHC class I (GO:0042590)	44	+	4.53E-03
regulation of cellular amide metabolic process (GO:0034248)	137	+	9.93E-03
positive regulation of protein ubiquitination involved in ubiquitin-dependent protein catabolic process (GO:2000060)	57	+	1.93E-02
GO cellular component complete			
small ribosomal subunit (GO:0015935)	36	+	0.00E+00
cytosolic ribosome (GO:0022626)	89	+	0.00E+00
large ribosomal subunit (GO:0015934)	51	+	0.00E+00
extracellular vesicle (GO:1903561)	897	+	1.62E-13
extracellular region (GO:0005576)	1017	+	9.97E-11
pigment granule (GO:0048770)	61	+	2.89E-05
cell-substrate adherens junction (GO:0005924)	202	+	4.31E-05
DNA packaging complex (GO:0044815)	18	+	6.35E-05
proteasome accessory complex (GO:0022624)	21	+	1.34E-02
ribonucleoprotein granule (GO:0035770)	60	+	2.21E-02

Supplementary Table S7. Manually curated list of Gene Ontology (GO) terms used for detecting ‘miRNA related’ proteins in the HEK-, C2C12 EVs and Vesiclepedia datasets.

GO term	GO Term description
GO:0010267	production of ta-siRNAs involved in RNA interference
GO:0010445	nuclear dicing body
GO:0010586	miRNA metabolic process
GO:0010587	miRNA catabolic process
GO:0016442	RISC complex
GO:0031053	primary miRNA processing
GO:0033391	chromatoid body
GO:0035068	micro-ribonucleoprotein complex
GO:0035195	gene silencing by miRNA
GO:0035196	production of miRNAs involved in gene silencing by miRNA
GO:0035198	miRNA binding
GO:0035278	miRNA mediated inhibition of translation
GO:0035279	mRNA cleavage involved in gene silencing by miRNA
GO:0035280	miRNA loading onto RISC involved in gene silencing by miRNA
GO:0035281	pre-miRNA export from nucleus
GO:0044747	mature miRNA 3'-end processing
GO:0044748	3'-5'-exoribonuclease activity involved in mature miRNA 3'-end processing
GO:0060964	regulation of gene silencing by miRNA
GO:0060965	negative regulation of gene silencing by miRNA
GO:0061614	pri-miRNA transcription from RNA polymerase II promoter
GO:0061715	miRNA 2'-O-methylation
GO:0061716	miRNA export from nucleus
GO:0061717	miRNA transporter activity
GO:0070578	RISC-loading complex
GO:0070877	microprocessor complex
GO:0070878	primary miRNA binding
GO:0090624	endoribonuclease activity. cleaving miRNA-paired mRNA
GO:0098806	deadenylation involved in gene silencing by miRNA
GO:0098851	double-stranded miRNA binding
GO:1903798	regulation of production of miRNAs involved in gene silencing by miRNA
GO:1903799	negative regulation of production of miRNAs involved in gene silencing by miRNA
GO:1903800	positive regulation of production of miRNAs involved in gene silencing by miRNA
GO:1905616	regulation of miRNA mediated inhibition of translation
GO:1905617	negative regulation of miRNA mediated inhibition of translation
GO:1905618	positive regulation of miRNA mediated inhibition of translation
GO:1990428	miRNA transport
GO:1990684	protein-lipid-RNA complex
GO:1990729	primary miRNA modification
GO:1990744	primary miRNA methylation
GO:2000625	regulation of miRNA catabolic process
GO:2000626	negative regulation of miRNA catabolic process
GO:2000627	positive regulation of miRNA catabolic process
GO:2000628	regulation of miRNA metabolic process
GO:2000629	negative regulation of miRNA metabolic process
GO:2000630	positive regulation of miRNA metabolic process
GO:2000634	regulation of primary miRNA processing
GO:2000635	negative regulation of primary miRNA processing
GO:2000636	positive regulation of primary miRNA processing
GO:2000637	positive regulation of gene silencing by miRNA
GO:0070883	pre-miRNA binding

Supplementary Table S8. HEK and C2C12 EV proteins and their respective 'miRNA related' protein ranks. Manually curated list of Gene Ontology (GO) terms describing miRNA-related cellular components, -molecular functions or -biological processes (Supplementary Table S7) was taken as a basis of identifying 'miRNA related' proteins and their abundance ranks. The ranks denote the abundance of the protein in the respective proteome (lowest rank=highest abundance).

Gene Ontology class	HEK EVs		C2C12 EVs	
	Gene ID	#Rank	Gene ID	#Rank
GO:0016442 RISC complex	EIF4E	#491	Snd1	#631
	SND1	#720	Eif4e	#706
	DDX6	#932	Ddx6	#896
	DICER1	#2249		
GO:0070578 RISC-loading complex	NSUN2	#331	Eif4e	#706
	EIF4E	#491	Nsun2	#1391
GO:0035068 micro-ribonucleoprotein complex	DICER1	#2249		
GO:0035198 miRNA binding	FMR1	#509	Fmr1	#1315
	HNRNPA2B1	#1503	Hnrnpa2b1	#1759
	RBM4	#2224.5		
	DICER1	#2249		
GO:1990428 miRNA transport	HNRNPA2B1	#1503	Hnrnpa2b1	#1759
GO:0010586 miRNA metabolic process	ZCCHC11	#2429		
GO:0010587 miRNA catabolic process	LIN28B	#1864.5		
	ZCCHC11	#2429		
GO:0035195 gene silencing by miRNA	EIF6	#872	Mov10	#786
	MOV10	#1029		
	CNOT1	#1673		
GO:0031053 primary miRNA processing	SRRT	#594.5	Mettl3	#205
	SMAD3	#1467	Smad2	#846
	HNRNPA2B1	#1503	Srrt	#1252
			Hnrnpa2b1	#1759
GO:1990744 primary miRNA methylation			Mettl3	#205
GO:0060964 regulation of gene silencing by miRNA	FMR1	#509	Eif4g1	#1251
	EIF4G1	#1104	Fmr1	#1315
	PUM1	#1176.5		
GO:2000628 regulation of miRNA metabolic process	DICER1	#2249		
	KHSRP	#2344		
GO:0035278 miRNA mediated inhibition of translation	EIF6	#872		
	RBM4	#2224.5		
GO:0060965 negative regulation of gene silencing by miRNA	ELAVL1	#961	Tgfb1	#1159
	TGFB1	#1602.5		
GO:2000630 positive regulation of miRNA metabolic process	HRAS	#1025	Hras	#592
	DICER1	#2249		
GO:2000637 positive regulation of gene silencing by miRNA	FMR1	#509	Fmr1	#1315
	FXR1	#733.5	FXR1	#1316
	PUM1	#1176.5		
GO:0035279 mRNA cleavage involved in gene silencing by miRNA	MOV10	#1029	Mov10	#786
GO:0035196 production of miRNAs involved in gene silencing by miRNA	PRKRA	#1476	Rbm3	#1807
	ADAR	#1548		
	LIN28B	#1864.5		
	DICER1	#2249		
	ZCCHC11	#2429		
GO:0035280 miRNA loading onto RISC involved in gene silencing by miRNA	ADAR	#1548		
	DICER1	#2249		
GO:1905618 positive regulation of miRNA mediated inhibition of translation	EIF4G1	#1104	Eif4g1	#1251
GO:1903799 negative regulation of production of miRNAs involved in gene silencing by miRNA	TGFB1	#1602.5	Tgfb1	#1159
			Ppp3ca	#1680
GO:1903800 positive regulation of production of miRNAs involved in gene silencing by miRNA	MAP2K2	#1126	Map2k1	#1189
	MAP2K1	#1306	Map2k2	#1296
	EGFR	#2555	Egfr	#1746.5
GO:0035281 pre-miRNA export from nucleus	RAN	#103	Ran	#62
	XPO5	#1204		
GO:0070883 pre-miRNA binding	RAN	#103	Ran	#62
	XPO5	#1204		
	DICER1	#2249		

Supplementary Table S9. Contribution of individual 'miRNA related' proteins to the total protein mass in HEK EVs. Details regarding the protein classifications under respective Gene Ontology (GO) terms can be found in Supplementary Table S8.

Gene symbol	MS Area	Total MS Area (%)	# of GO terms including the protein	Protein contribution to GO-related analysis (%)
EIF4E	4.80E+08	0.026	2	0.052
SND1	2.61E+08	0.014	1	0.014
DDX6	1.74E+08	0.009	1	0.009
DICER1	2.95E+07	0.002	8	0.013
NSUN2	8.14E+08	0.044	1	0.044
FMR1	4.50E+08	0.024	3	0.073
HNRNPA2B1	7.82E+07	0.004	3	0.013
ZCCHC11	1.99E+07	0.001	3	0.003
EIF6	1.93E+08	0.010	2	0.021
MOV10	1.45E+08	0.008	2	0.016
CNOT1	6.42E+07	0.003	1	0.003
SRRT	3.56E+08	0.019	1	0.019
SMAD3	8.17E+07	0.004	1	0.004
EIF4G1	1.29E+08	0.007	2	0.014
PUM1	1.18E+08	0.006	2	0.013
KHSRP	2.42E+07	0.001	1	0.001
RBM4	3.06E+07	0.002	2	0.003
ELAVL1	1.63E+08	0.009	1	0.009
TGFB1	6.91E+07	0.004	2	0.007
HRAS	1.45E+08	0.008	1	0.008
FXR1	2.54E+08	0.014	1	0.014
PRKRA	8.03E+07	0.004	1	0.004
ADAR	7.39E+07	0.004	2	0.008
LIN28B	4.95E+07	0.003	2	0.005
MAP2K2	1.26E+08	0.007	1	0.007
MAP2K1	9.90E+07	0.005	1	0.005
EGFR	1.07E+07	0.001	1	0.001
RAN	4.60E+09	0.249	2	0.498
XPO5	1.14E+08	0.006	2	0.012
**SUM**		**0.500**		**0.895**

Supplementary Table S10. Contribution of individual 'miRNA related' proteins to the total protein mass in C2C12 EVs. Details regarding the protein classifications under respective Gene Ontology (GO) terms can be found in Supplementary Table S8.

Gene symbol	MS Area	Total MS Area (%)	# of GO terms including the protein	Protein contribution to GO-related analysis (%)
Snd1	8.65E+07	0.020	1	0.020
Eif4e	7.10E+07	0.016	2	0.032
Ddx6	4.53E+07	0.010	1	0.010
Nsun2	1.67E+07	0.004	1	0.004
Fmr1	1.91E+07	0.004	3	0.013
Hnrnpa2b1	5.54E+06	0.001	3	0.004
Mettl3	4.70E+08	0.108	2	0.215
Smad2	5.15E+07	0.012	1	0.012
Eif4g1	2.14E+07	0.005	2	0.010
Tgfb1	2.68E+07	0.006	2	0.012
Hras	9.45E+07	0.022	1	0.022
Fxr1	1.91E+07	0.004	1	0.004
Rbm3	3.53E+06	0.001	1	0.001
Ppp3ca	7.97E+06	0.002	1	0.002
Map2k1	2.47E+07	0.006	1	0.006
Map2k2	1.97E+07	0.004	1	0.004
Egfr	6.07E+06	0.001	1	0.001
Ran	1.10E+09	0.252	2	0.505
Mov10	5.84E+07	0.013	2	0.027
Srrt	2.14E+07	0.005	1	0.005
**SUM**		**0.497**		**0.909**

Supplementary Table S11. Proteins in RNA binding Gene Ontology subclasses contributing most to the proteomes of HEK and C2C12 EVs. Top three proteins classified under RNA binding protein (GO:0003723) Gene Ontology subclasses that make up more than 1% of the EV proteome are depicted. Grey-shaded entries of C2C12 EVs denote the GO which contributed <1% of the C2C12 EV proteome.

**Gene Ontology classification**	**HEK EVs**			**C2C12 EVs**		
	**Gene ID**	**Protein identifier**	**Total MS Area (%)**	**Gene ID**	**Protein identifier**	**Total MS Area (%)**
GO:0044822 poly(A) RNA binding	RPS8	ENSP00000379888	1.13	Rps27a	ENSMUSP00000099909	1.37
	RPS3	ENSP00000434643	0.92	Mrps26	ENSMUSP00000123324	1.35
	RPS3	ENSP00000416745	0.80	Hspa8	ENSMUSP00000015800	0.97
GO:0019843 rRNA binding	RPS3	ENSP00000434643	0.92	Rps3	ENSMUSP00000032998	0.42
	RPS3	ENSP00000416745	0.80	Rps9	ENSMUSP00000006496	0.23
	RPS9	ENSP00000478449	0.78	Rps4x	ENSMUSP00000033683	0.21
GO:0008135 translation factor activity. RNA binding	EEF1A1	ENSP00000339063	0.41	Eef1a1	ENSMUSP00000042457	0.59
	EEF1A2	ENSP00000298049	0.24	Eef1a2	ENSMUSP00000054556	0.40
	EEF2	ENSP00000307940	0.14	Eef2	ENSMUSP00000046101	0.13
GO:0003725 double-stranded RNA binding	TUBB4B	ENSP00000341289	0.70	Slc3a2	ENSMUSP00000010239	0.29
	HSP90AB1	ENSP00000360709	0.18	Tuba1b	ENSMUSP00000076777	0.26
	CLTC	ENSP00000376763	0.13	Hsp90ab1	ENSMUSP00000024739	0.20
GO:0003727 single-stranded RNA binding	HNRNPU	ENSP00000393151	0.31	Anxa1	ENSMUSP00000025561	0.06
	HNRNPC	ENSP00000450629	0.25	Pabpc1	ENSMUSP00000001809	0.04
	HNRNPC	ENSP00000451291	0.25	Pabpc4	ENSMUSP00000077794	0.03

REFERENCES

1. Wei, Z. *et al.* Fetal Bovine Serum RNA Interferes with the Cell Culture derived Extracellular RNA. *Sci. Rep.* **6,** 31175 (2016).
